# Replication of multiple system atrophy prions in primary astrocyte cultures from transgenic mice expressing human α-synuclein

**DOI:** 10.1186/s40478-019-0703-9

**Published:** 2019-05-20

**Authors:** Zuzana Krejciova, George A. Carlson, Kurt Giles, Stanley B. Prusiner

**Affiliations:** 1 Institute for Neurodegenerative Diseases, UCSF Weill Institute for Neurosciences, University of California, San Francisco, Sandler Neurosciences Center, 675 Nelson Rising Lane, San Francisco, CA 94158 USA; 2 Department of Neurology, UCSF Weill Institute for Neurosciences, University of California, San Francisco, San Francisco, CA 94158 USA; 3 Department of Biochemistry and Biophysics, University of California, San Francisco, San Francisco, CA 94158 USA

**Keywords:** MSA, Prion, Astrocytes, α-Synuclein, Proteinopathies

## Abstract

Glial cytoplasmic inclusions (GCIs) containing aggregated and hyperphosphorylated α-synuclein are the signature neuropathological hallmark of multiple system atrophy (MSA). Native α-synuclein can adopt a prion conformation that self-propagates and spreads throughout the brain ultimately resulting in neurodegeneration. A growing body of evidence argues that, in addition to oligodendrocytes, astrocytes contain α-synuclein inclusions in MSA and other α-synucleinopathies at advanced stages of disease. To study the role of astrocytes in MSA, we added MSA brain homogenate to primary cultures of astrocytes from transgenic (Tg) mouse lines expressing human α-synuclein. Astrocytes from four Tg lines, expressing either wild-type or mutant (A53T or A30P) human α-synuclein, propagated and accumulated α-synuclein prions. Furthermore, we found that MSA-infected astrocytes formed two morphologically distinct α-synuclein inclusions: filamentous and granular. Both types of cytoplasmic inclusions shared several features characteristic of α-synuclein inclusions in synucleinopathies: hyperphosphorylation preceded by aggregation, ubiquitination, thioflavin S–positivity, and co-localization with p62. Our findings demonstrate that human α-synuclein forms distinct inclusion morphologies and propagates within cultured Tg astrocytes exposed to MSA prions, indicating that α-synuclein expression determines the tropism of inclusion formation in certain cells. Thus, our work may prove useful in elucidating the role of astrocytes in the pathogenic mechanisms that feature in neurodegeneration caused by MSA prions.

**Electronic supplementary material:**

The online version of this article (10.1186/s40478-019-0703-9) contains supplementary material, which is available to authorized users.

## Introduction

The aggregation and intracellular deposition of α-synuclein in neurons or glia define a group of neurodegenerative disorders called α-synucleinopathies. In the late 1990s, α-synuclein was genetically linked to Parkinson’s disease (PD) when autosomal dominant mutations in *SNCA* were discovered [40, 72]. Interest in the protein intensified when it was found to be a major component of Lewy bodies, the intracellular aggregates found in all PD patients [84]. Inclusion bodies present in individuals with other α-synucleinopathies, including multiple system atrophy (MSA), also contain α-synuclein [57, 68, 84]. While neurons containing Lewy bodies and Lewy neurites are distinctive features of PD and dementia with Lewy bodies (DLB), α-synuclein-positive inclusions have also been found in postmortem PD brains in astrocytes and oligodendrocytes [8, 32, 95]. The pathognomonic hallmark of MSA is the presence of α-synuclein aggregates in white matter oligodendrocytes called glial cytoplasmic inclusions (GCIs) ([83, 91, 96]). Moreover, α-synuclein-positive astroglial inclusions have been found in MSA brains, however, in a lower density compared with GCIs [100].

Pathological protein aggregates directly affecting neurons are often thought to be the sole cause of neurodegenerative diseases; however, this supposition has not been unequivocally demonstrated. Notably, astrocytes are the most abundant glial cell type, and glia comprise more than half of the total brain cells in mammals, secreting regulatory cues in every stage of synapse development [16, 86, 94]. Glia play a crucial role in maintaining neuronal homeostasis, which is likely disrupted by an accumulation of pathological protein. It is thought that homeostatic failure is often a primary cause of disease [37, 49, 53, 93]. We investigated pathogenic α-synuclein aggregation in astrocytes to understand its role in disease. Although astrocytes normally express little α-synuclein, accumulation of abnormally phosphorylated and aggregated α-synuclein has been shown in subpial and periventricular astrocytes in ~ 40% of patients with MSA [62] and in all patients with DLB and with sporadic PD at stage 4 [8].

Recent studies in mice have demonstrated that α-synuclein from MSA patient samples can replicate by templated self-propagation and spread—i.e., it becomes a prion [71, 74, 99, 102]. Here, we show that primary astrocytes isolated from transgenic (Tg) mice propagate MSA prions and accumulate α-synuclein inclusions when exposed to α-synuclein fibrils or MSA human brain homogenates. For our initial studies, we chose the TgM83 mouse line, which overexpresses human α-synuclein with the familial PD A53T mutation [28]. This line develops α-synuclein pathology in astrocytes and neurons following inoculation with α-synuclein fibrils [52]. After exposing cultured astrocytes to wild-type (wt) α-synuclein fibrils, we evaluated α-synuclein aggregation and phosphorylation at Ser129, here abbreviated pSyn (S129), as phosphorylation of α-synuclein at this residue is a major indicator of α-synucleinopathy. In the exposed astrocytes, the exogenously added fibrils efficiently induced dose- and gene-dose-dependent aggregation and phosphorylation of endogenously expressed human α-synuclein.

We next exposed TgM83 astrocytes to (1) MSA patient brain homogenates, (2) Tg mouse brain in which MSA had been passaged, and (3) cell homogenate from MSA-infected astrocyte cultures. After exposing the cultured astrocytes to each of these MSA prions, we found that α-synuclein aggregation and phosphorylation was rapid, progressive, and dose-dependent.

Our findings demonstrate that α-synuclein inclusions form within cultured astrocytes exposed to MSA prions and thus may prove useful in elucidating the contribution of astrocytes to the pathogenic mechanisms that feature in neurodegeneration. Importantly, our studies provide a useful cell culture model to understand various pathological forms of α-synuclein prions in astrocytes and how they contribute to disease at the cellular and subcellular levels. Our culture system may also provide a novel screening platform for discovering therapeutics against MSA and other α-synucleinopathies.

## Results

### Differences in α-synuclein expression in astrocytes from four Tg mouse lines

Primary cultures of astrocytes from cortical brain tissue from Tg mice 1–4 days old (Additional file 1: Figure S1A) were composed of ~ 90% astrocytes expressing the extracellular astrocyte-specific transmembrane glutamate-aspartate transporter marker GLAST (Additional file 1: Figure S1B,C). The TgM83 mouse line, which expresses mutant human α-synuclein (A53T) under control of the prion protein gene (*Prnp*) promoter, was selected for a majority of our studies. TgM83^+/+^ and TgM83^+/−^ astrocytes express α-synuclein in a gene-dose-dependent manner—i.e., astrocytes from homozygous mice express approximately 2-fold more α-synuclein than cells from hemizygous animals (Additional file 1: Figure S2A,B).

In the other three Tg mouse lines that express human α-synuclein under control of its own regulatory sequences in a P1 artificial chromosome (PAC), slight differences in astrocytic α-synuclein expression were apparent. The Tg(SNCA*A53T)Nbm astrocytes expressed the least α-synuclein, whereas the Tg(*SNCA*^+/+^)Nbm line expressed the most (Additional file 1: Figure S2A,B), in contrast to whole brain relative expression patterns (Additional file 1: Figure S2C,D). Notably, TgM83 lines express both human and endogenous mouse α-synuclein, while the TgNbm lines express human α-synuclein on a murine α-synuclein knockout background. We confirmed that astrocytes and brain tissue homogenates from control *Snca* knockout mice do not react with human-specific α-synuclein antibodies. Immunofluorescent analysis confirmed expression of human α-synuclein in a punctate pattern in astrocytes from all Tg lines (Additional file 1: Figure S2E).

### Exogenously added recombinant α-synuclein fibrils induce aggregation and phosphorylation of endogenously expressed α-synuclein in TgM83 astrocytes

To determine the role of astrocytes in MSA and other α-synuclein proteinopathies, it was first necessary to evaluate whether primary astrocytes from Tg mice overexpressing human α-synuclein could generate aggregated and phosphorylated α-synuclein. Primary cultures of astrocytes from cortical brain tissue from Tg mice 1–4 days old were examined for human α-synuclein expression using the human-specific Syn211 antibody.

We confirmed the absence of spontaneous accumulation of phosphorylated α-synuclein in all unexposed control cultures by immunostaining with anti-phosphorylated α-synuclein antibody. Immunostaining with anti-glial fibrillary acidic protein (GFAP) antibody confirmed the astrocytic phenotype of the cells in the cultures. After confirming α-synuclein expression in our cell cultures, we asked whether primary Tg mouse astrocytes could form aggregates of phosphorylated α-synuclein. To address this question, we exposed TgM83^+/+^ astrocytes to culture medium containing 10 μg/mL recombinant wt α-synuclein fibrils for 48 h. We observed rapid uptake of fibrils and robust intracellular aggregation of hyperphosphorylated α-synuclein within the GFAP-positive astrocytes by 7 days post-exposure (dpe), which continued to increase to 21 dpe (Additional file 1: Figure S3A). We showed this increase to be a function of time. Moreover, TgM83^+/+^ astrocytes exposed to 40 μg/mL of recombinant wt α-synuclein fibrils exhibited the highest amount of aggregates at the latest time point. TgM83^+/+^ cultures exposed to 10 μg/mL of recombinant wt α-synuclein fibrils had fewer aggregates, and cultures exposed to 2.5 μg/mL had the least. We observed the same pattern of aggregate formation in the hemizygous cultures, although at substantially lower levels (Additional file 1: Figure S3B).

We next exposed TgM83^+/+^ astrocytes to medium containing Alexa Fluor 488–conjugated α-synuclein fibrils for 48 h. The cultures were then extensively washed and further cultured in fresh media for 7 days. We observed small aggregates of α-synuclein phosphorylated at serine 129 (S129) at 48 h post-exposure (Additional file 1: Figure S3B). At 7 dpe, juxtanuclear aggregates of phosphorylated α-synuclein were detected (Additional file 1: Figure S3C). We further observed that the exogenously added Alexa Fluor 488–conjugated α-synuclein fibrils entered the cells and efficiently induced aggregation and phosphorylation of endogenously expressed α-synuclein in the exposed astrocytes.

### Human MSA prions replicate in cultured Tg mouse astrocytes

**Fig. 1:**
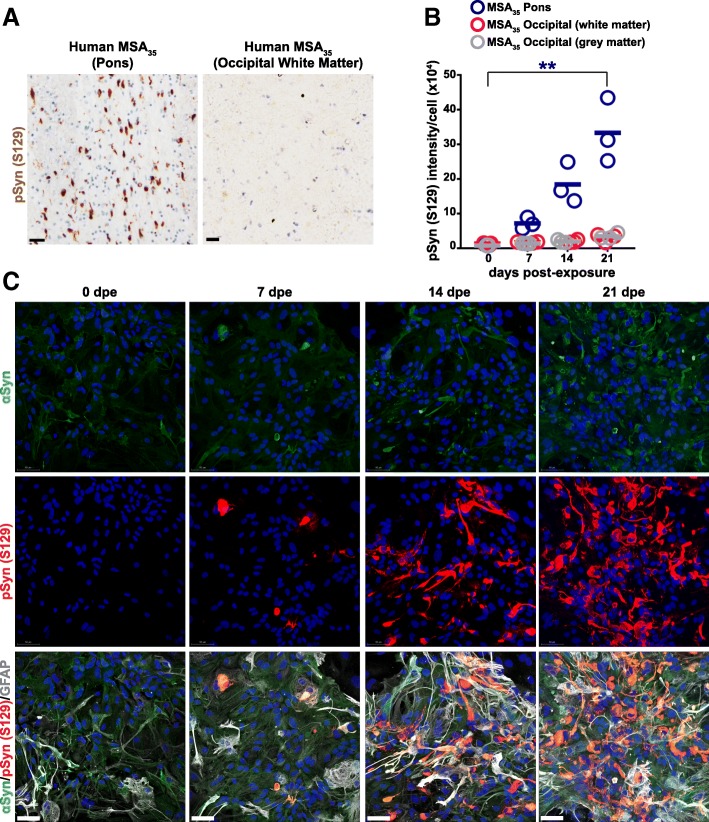
α-Synuclein accumulation and phosphorylation at serine 129 in primary astrocytes exposed to human MSA brain homogenate. **a** Representative images of immunohistochemical detection of α-synuclein deposits in a patient sample, MSA_35_. Brain tissue from pons (left) and occipital cortex (right). Tissues were immunostained for phosphorylated α-synuclein at serine 129 [pSyn (S129), brown], and nuclei are in blue. Scale bars, 100 μm. **b** Quantification of pSyn (S129) signal intensity in TgM83^+/+^ astrocytes exposed to MSA_35_ brain tissue from pons (dark blue circles), occipital cortex (white matter; pink circles), and occipital cortex (grey matter; grey circles). Only tissue from the midbrain (pons) region induced rapid accumulation of pSyn (S129) over time. The signal intensity was normalized by cell count. Data are plotted with mean (*n* = 3) and analyzed using an unpaired *t-*test. **c** Representative immunographs of primary TgM83^+/+^ astrocytes exposed to 0.5% MSA_35_ brain homogenate (pons) for 48 h and further cultured in fresh media up to 21 days post-exposure (dpe). Cells were immunostained for total human α-synuclein (αSyn, green), pSyn (S129) (red), and glial fibrillary acidic protein (GFAP, white). Merge of all three channels is shown in the bottom row. Nuclei were stained with DAPI (blue). Scale bars, 50 μm

Given the successful transmission of MSA prions to TgM83 mice expressing human α-synuclein with the A53T mutation [74, 99], we exposed primary astrocyte cultures isolated from these animals to MSA patient brain homogenates (Fig. 1). Based on the Braak model [7], we prepared homogenates from two brain regions: the pons and occipital cortex. Homogenate prepared from the pons contained abundant phosphorylated α-synuclein; conversely, the occipital cortex displayed a much lower amount (Fig. 1a). We exposed TgM83^+/+^ astrocytes to 0.5% MSA brain homogenates from the pons and occipital cortex (both white and grey matter) for 48 h. The cells were recovered by two DPBS washes and further cultured up to 21 dpe. We observed a progressive accumulation of phosphorylated α-synuclein in astrocyte cultures exposed to homogenate from the pons (Fig. 1b). However, neither grey nor white matter homogenates from the occipital cortex induced α-synuclein aggregates at levels different from controls. In astrocytes (GFAP, white) exposed to homogenate from the pons, immunocytochemistry confirmed rapid, robust, and time-dependent aggregation of phosphorylated α-synuclein [total αSyn (Syn211), green; pSyn (S129), red] that accumulated in the cytoplasm and formed large juxtanuclear inclusions (Fig. 1c).

### Transmission of TgM83-passaged MSA prions to astrocytes

**Fig. 2:**
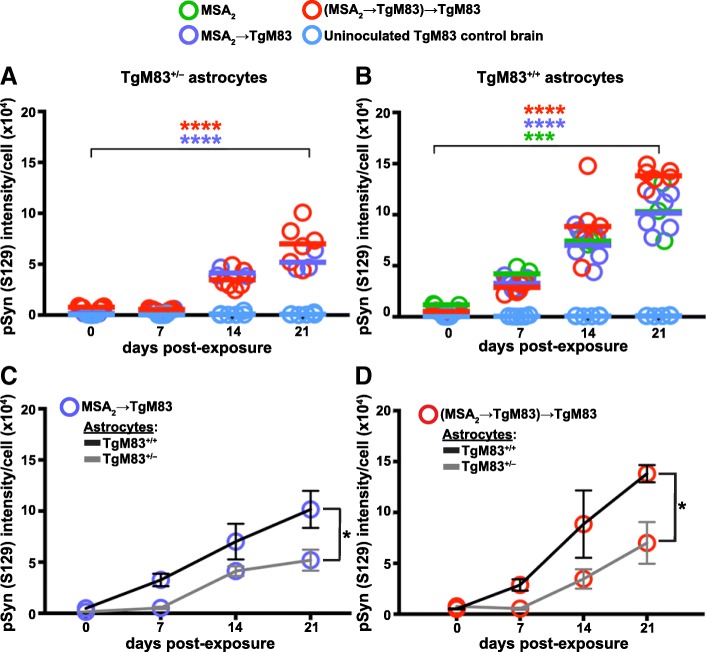
α-Synuclein aggregation and inclusion formation in MSA-exposed TgM83 astrocytes is rapid, gene-dose-, and time-dependent. Quantification of **a** TgM83^+/−^ and **b** TgM83^+/+^ astrocyte cultures exposed to 0.5% brain homogenates from MSA_2_ (green circles, *n* = 3), primary passaged MSA_2_ in TgM83^+/−^ mice (purple circles, *n* = 3 TgM83^+/−^ astrocytes; *n* = 6 TgM83^+/+^ astrocytes), secondary passaged MSA_2_ in TgM83^+/−^ mice (red circles, *n* = 6), and age-matched control TgM83^+/+^ littermates (blue circles, *n* = 5). Cultures were analyzed at 0, 7, 14, and 21 days post-exposure (dpe). Astrocytes were immunostained for phosphorylated α-synuclein (S129), and the signal intensity was normalized by cell count. We observed a rapid, gene-dose-, and time-dependent accumulation of phosphorylated α-synuclein in astrocytes exposed to MSA prions. Data are plotted with mean and analyzed by one-way ANOVA followed by Tukey’s multicolumn comparison test: ****, *P* < 0.0001; ***, *P* = 0.0003. **c**, **d** Gene-dose dependency was analyzed by plotting data from (**a**, **b**) where TgM83^+/+^ (black line) and TgM83^+/−^ (grey line) astrocyte cultures were exposed to (**c**) primary passaged MSA_2_ (purple circles). Linear regression was applied to each group (TgM83^+/+^, *P* = 0.0015; TgM83^+/−^, *P* = 0.0496) followed by ANCOVA analysis of covariance to compare the slopes: *, *P* = 0.0334; F = 10.13; DF*n* = 1; DFd = 4. **d** The same Tg mouse lines were exposed to secondary passaged MSA_2_ (red circles). Linear regression was applied to each group (TgM83^+/+^, *P* = 0.0130; TgM83^+/−^, *P* = 0.0736) followed by ANCOVA analysis of covariance to compare the slopes: *, *P* = 0.0411; F = 8.82; DFn = 1; DFd = 4

We observed that MSA prion accumulation was less extensive in the transgene hemizygous astrocytes compared with the homozygous cells (Fig. 2). We exposed TgM83^+/+^ and TgM83^+/−^ cultured astrocytes to MSA brain homogenate from primary (purple circles, Fig. 2) and secondary passage (red circles, Fig. 2) in TgM83^+/−^ mice. In both cases, significantly higher levels of α-synuclein inclusions accumulated in the homozygous, compared with the hemizygous, astrocytes (Fig. 2c, d). Brain homogenate (0.5%) from an age-matched, uninoculated TgM83^+/+^ mouse did not induce α-synuclein aggregation or phosphorylation in astrocyte cultures from either line (light blue circles, Fig. 2a, b). This finding further argues that the level of accumulation of phosphorylated α-synuclein inclusions in astrocytes is dependent on the level of transgene-encoded α-synuclein protein in cells exposed to MSA brain homogenate. The aggregated state of α-synuclein forming inclusions in cultures exposed to MSA brain homogenate was confirmed by immunostaining with the amyloid detecting dye, thioflavin S (Additional file 1: Figure S4). Representative photomicrographs of immunocytochemistry of TgM83^+/+^ astrocytes exposed to second-passage MSA brain homogenate are shown in Additional file 1: Figure S5.

### Passage of MSA prions in cultured TgM83 astrocytes

**Fig. 3:**
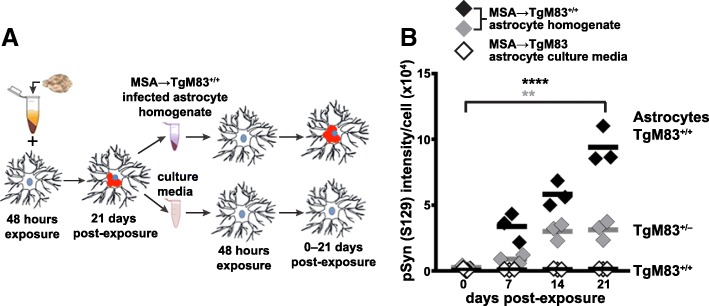
Subpassage of MSA prions in TgM83 cultured astrocytes. **a** Schematic representation of cell homogenate preparation and media collection from MSA-infected TgM83^+/+^ astrocyte cultures at 21 dpe. Naïve astrocytes were then exposed to the cell homogenate or medium for 48 h, cultured up to 21 days, and analyzed for pSyn (S129) at four time points. **b** Quantification of TgM83^+/+^ (black diamonds) and TgM83^+/−^ (grey diamonds) astrocytes exposed to MSA-infected cell homogenate and TgM83^+/+^ astrocytes cultured with media previously cultured with MSA-infected astrocytes (white diamonds). Astrocytes were immunolabeled for pSyn (S129), and the signal intensity was normalized by cell count. Data are plotted with mean and analyzed by one-way ANOVA followed by Tukey’s multicolumn comparison test: ****, *P* < 0.0001; **, *P* = 0.0017

A key feature of prions is their ability to initiate templated replication upon transmission to an uninfected host [73]. To investigate whether MSA prions that formed in TgM83^+/+^ primary astrocytes in vitro were able to infect naïve astrocytes, we used extracts of astrocyte cultures previously exposed to MSA brain homogenate and cultured for 21 days as inoculum for naïve TgM83^+/+^ or TgM83^+/−^ astrocyte cultures (Fig. 3a,b). We also exposed naïve TgM83^+/+^ astrocytes to culture media previously cultured with MSA-infected astrocytes to determine if infectivity could spread extracellularly. Exposure of naïve astrocytes to MSA-infected astrocytic culture homogenate resulted in robust, progressive MSA infection in both TgM83^+/+^ and TgM83^+/−^ astrocytes, confirming prion transmission (Fig. 3b). Moreover, we observed the effect of gene-dose-dependency on robustness of α-synuclein aggregation and phosphorylation. TgM83^+/+^ astrocytes exposed to MSA-infected cell inoculum harbored a higher amount of α-synuclein inclusions. However, the naïve TgM83^+/+^ astrocytes exposed to culture media previously cultured with MSA-infected astrocytes did not propagate MSA infection at a detectable level (Fig. 3b).

### α-Synuclein aggregates into two morphologically distinct inclusions: filamentous and granular

**Fig. 4:**
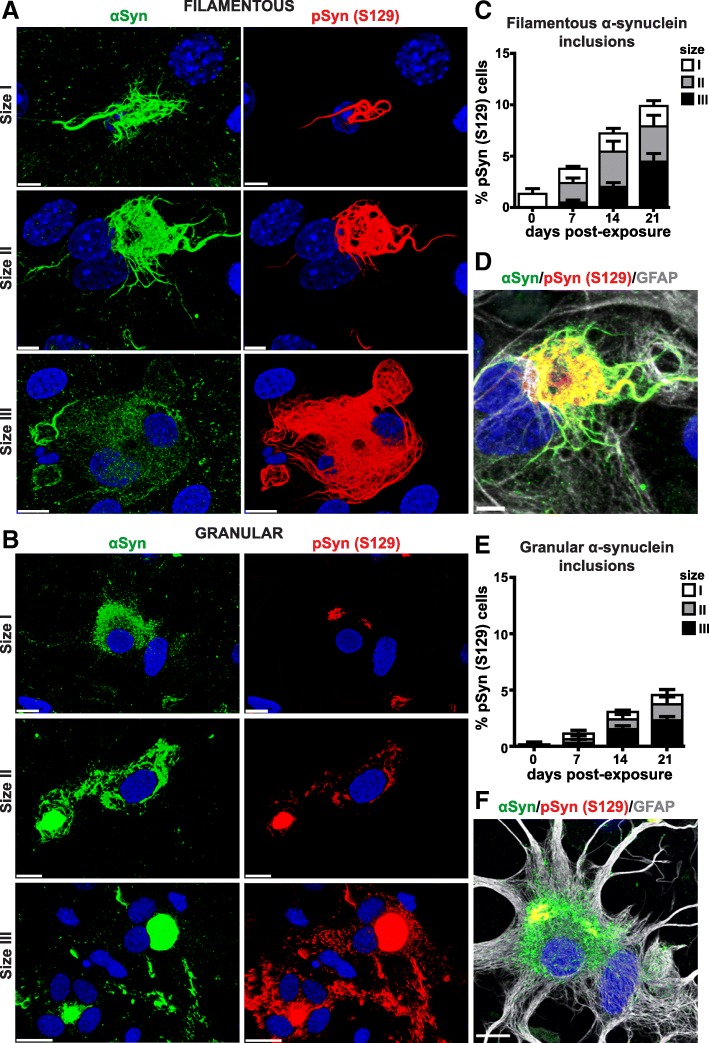
Filamentous and granular α-synuclein inclusions form in MSA-infected cultured TgM83 astrocytes. 3D volume visualization of maximum intensity projection of Z-stacks of α-synuclein inclusions in MSA-infected TgM83^+/+^ astrocytes aggregating as **a** filamentous and **b** granular α-synuclein inclusions. **c**, **e** Quantification of pSyn (S129) inclusions formed in MSA-infected TgM83^+/+^ cultures [size I (< 10 μm), size II (10–50 μm), and size III (> 50 μm)] at 0, 7, 14, and 21 days post-exposure (dpe). Cell count of each group is represented as percentage of total cells, and the data were acquired from 16 randomized fields from four replicate experiments (*n* = 4). Astrocytes were immunostained for total human α-synuclein (αSyn, green), phosphorylated α-synuclein [pSyn (S129), red], and GFAP (white). **d**, **f** Maximum projection intensity of Z-stack and merge of all three channels is shown. Co-localization of pSyn (S129) and αSyn appears yellow. Nuclei were stained with DAPI (blue). Scale bars, **a**, **b** 20 μm, **d** 5 μm, **f** 10 μm

Immunocytochemistry revealed that α-synuclein, which typically appears in a fine punctate pattern in control cells, aggregates into either filamentous (Fig. 4a,c, and d) or granular (Fig. 4b,e, and f) inclusions when exposed to MSA prions. We quantified inclusion size in MSA-infected TgM83^+/+^ astrocytes at four time points after immunostaining with anti-phosphorylated α-synuclein antibody. To reveal the dynamics of α-synuclein aggregation and phosphorylation, we divided the inclusions into three sizes: I (< 10 μm), II (10–50 μm), and III (> 50 μm). Data from independent replicates (*n* = 4) were analyzed and plotted (Fig. 4c,e). The majority of inclusions at 0 dpe were size I (< 10 μm); these inclusions were present at each time point in roughly the same amount, suggesting a persistent formation of new inclusions. Inclusions at 7 dpe were mostly sizes I (~ 1.5% of cells) and II (~ 2%). Size III (> 50 μm) inclusions became evident over time and were most prominent at 21 dpe, at which point almost half of the MSA-infected cells (~ 15% of total cell count) contained inclusions of this size (Fig. 4c,e). Filamentous inclusions were more abundant (~ 10% of total number of inclusions independent of size) (Fig. 4c) than their granular counterparts (5%) at 21 dpe (Fig. 4e).

During the initial days post-exposure, staining with antibodies against unphosphorylated α-synuclein aggregates (green) were more apparent in inclusions than S129 phosphorylated α-synuclein immunostaining (red) (size I; Fig. 4a). However, by the time the inclusions reached > 50 μm in size, the majority of aggregated α-synuclein was phosphorylated (size III; Fig. 4a). Strikingly, any individual MSA-infected cell contained either filamentous (Fig. 4d; Additional file 1: Figure S6A) or granular (Fig. 4f; Additional file 1: Figure S6B) inclusions, but never both types together. Notably, both filamentous (Fig. 4d) and granular (Fig. 4f) inclusions were within the intracellular cytoplasmic compartment of the GFAP-labeled astrocytes (white). By using an additional astrocytic marker, the glutamate-aspartate transporter, GLAST (cyan), we confirmed that both inclusion types form in cells of astrocytic phenotype (Additional file 1: Figure S6).

### Biochemical hallmarks of synucleinopathies are recapitulated in cultured astrocytes propagating MSA prions

**Fig. 5:**
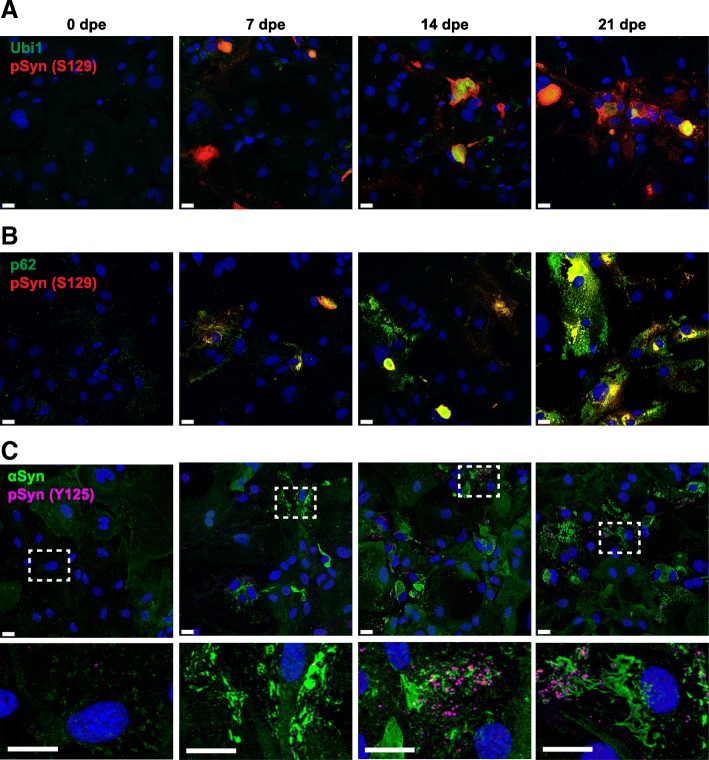
α-Synuclein inclusions in MSA-infected astrocytes are ubiquitinated and co-localized with p62 or contain α-synuclein phosphorylated at Y125. Representative immunographs of TgM83^+/+^ primary astrocytes exposed to 0.5% secondary passaged MSA_2_ brain homogenate for 48 h and immunostained at 0, 7, 14, and 21 days post-exposure (dpe) for **a** ubiquitin (green) and pSyn (S129) (red), **b** p62 (green) and pSyn (S129) (red), and **c** αSyn (green) and pSyn Y125 (magenta). Merge channels are shown, and **c** insets of dashed areas are shown in the bottom row. Nuclei were stained with DAPI (blue). Scale bars, 20 μm

To determine whether additional pathological hallmarks of synucleinopathies were recapitulated in our TgM83 astrocyte cultures, we examined the co-localization of aggregated and phosphorylated α-synuclein with ubiquitin and the ubiquitin-binding protein p62 (also known as sequestosome-1). We exposed TgM83 astrocytes to MSA brain homogenates using the same paradigm as described above. We analyzed MSA-infected astrocytes at four time points and found that α-synuclein inclusions co-localized with ubiquitin (Fig. 5a) and were heavily labeled with a p62 marker (Fig. 5b). In MSA-infected cells, p62 marker expression increased with aggregation of phosphorylated α-synuclein over 21 dpe, whereas both remained constant in the unexposed control cells (Additional file 1: Figure S7).

Moreover, we immunostained MSA-infected TgM83 astrocytes with Syn211 to determine the total α-synuclein (green, Fig. 5c) and phosphorylated α-synuclein at Y125 (magenta, Fig. 5c). Within α-synuclein inclusions, we detected a punctate pattern of α-synuclein phosphorylated at this site (Fig. 5c), which appeared more robust at later time points after exposure to MSA inoculum (14 and 21 dpe).

### MSA prions replicate in cultured astrocytes expressing wt, A53T, or A30P α-synuclein

**Fig. 6:**
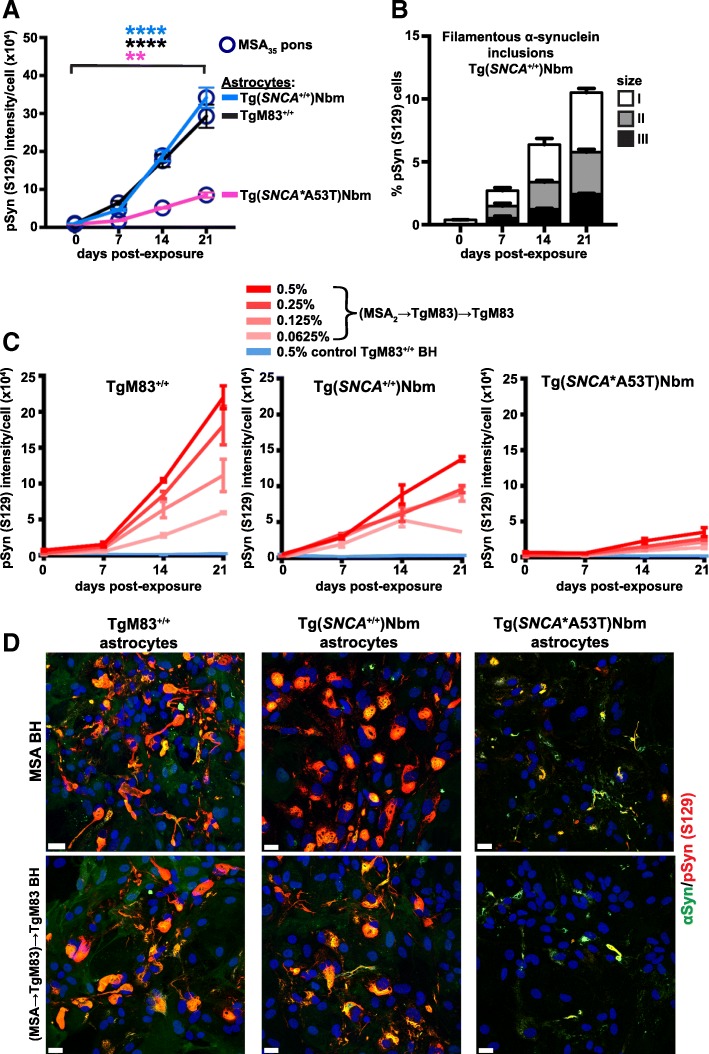
Differing intensity of MSA prion infection in astrocytes expressing wt and human mutant α-synuclein (A53T). **a** Quantification of signal intensity of aggregated α-synuclein inclusions [pSyn (S129)] in Tg(*SNCA*^+/+^)Nbm (light blue line), TgM83^+/+^ (black line), and Tg(SNCA*A53T)Nbm (magenta line) astrocytes exposed to 0.5% MSA_35_ brain homogenate (pons, dark blue circles). Data are plotted with mean and analyzed by one-way ANOVA followed by Tukey’s multicolumn comparison test: ****, *P* < 0.0001; **, *P* = 0.0076. **b** Quantification of pSyn (S129) inclusions formed in MSA-infected Tg(*SNCA*^+/+^)Nbm cultures [size I (< 10 μm), size II (10–50 μm), and size III (> 50 μm)] at 0, 7, 14, and 21 days post-exposure (dpe). Cell count of each group is represented as percentage of total cells, and the data were acquired from 16 randomized fields from two replicate experiments (*n* = 2). **c** Graphic representation of same lines of Tg astrocytes as in (**b**) exposed to 0.5, 0.25, 0.125, and 0.0625% TgM83-passaged MSA and 0.5% TgM83^+/+^ control littermate brain homogenate. **b**, **c** Cultures were immunostained for pSyn (S129) at 0, 7, 14, and 21 dpe, and the signal intensity was normalized by cell count. Data are plotted with mean **b** (*n* = 3–6) and **c** (*n* = 1). **d** Representative immunographs of primary astrocytes from (**b**, **c**) immunostained for human αSyn (green) and pSyn (S129) (red). Merged channels are shown. Nuclei were stained with DAPI (blue). Scale bars, 20 μm

We next compared susceptibility to MSA prion infection among TgM83^+/+^ astrocytes and Tg(*SNCA*^+/+^)Nbm and Tg(SNCA*A53T)Nbm mouse lines (Fig. 6). Human α-synuclein expression was lower in the wt cultures than in cultures from TgM83^+/+^ and Tg(SNCA*A53T)Nbm mice, the latter of which expresses the least human α-synuclein among the three lines. This finding indicates that less α-synuclein is available as a substrate for prion propagation (Additional file 1: Figure S2A, B, and E). We exposed the three lines to 0.5% MSA patient brain homogenate and found different efficiencies of aggregation and formation of phosphorylated α-synuclein (Fig. 6a). These lines express α-synuclein on either mouse *Snca* wt (TgM83) or knockout backgrounds [Tg(*SNCA*^+/+^)Nbm, Tg(SNCA*A53T)Nbm, and Tg(SNCA*A30P)Nbm]. Nevertheless, TgM83^+/+^ and Tg(*SNCA*^+/+^)Nbm astrocytes overexpressing either α-synuclein with the A53T mutation or wild-type, respectively, had similarly formed inclusions of phosphorylated α-synuclein.

A very low amount of α-synuclein inclusions accumulated in the Tg(SNCA*A53T)Nbm culture (Fig. 6a). This phenomenon recurred when astrocytes from the three lines were exposed to MSA brain homogenate passaged in TgM83 mice at four different concentrations. Accumulation of α-synuclein inclusions is dose-dependent: higher concentrations of MSA brain homogenate induced more phosphorylated α-synuclein inclusions in the culture (Fig. 6c). Furthermore, the amino acid sequence of the MSA_35_ brain homogenate is wild-type, which is the same as the α-synuclein expressed in the Tg(*SNCA*^+/+^)Nbm astrocytes on an *Snca* knockout background. In contrast, the TgM83-passaged MSA homogenate was passaged in mice expressing α-synuclein with the PD-linked A53T mutation on a mouse *Snca* wt background.

Interestingly, MSA-infected Tg(*SNCA*^+/+^)Nbm astrocytes expressing human wt α-synuclein on a murine *Snca* knockout background accumulated solely filamentous inclusions (Fig. 6b). However, the kinetics of α-synuclein inclusion formation and the proportion of MSA-infected astrocytes at 21 dpe was similar to that in MSA-infected TgM83^+/+^ astrocytes forming filamentous inclusions (Fig. 4c). Brain homogenate from a TgM83^+/+^ age-matched uninoculated control did not induce α-synuclein aggregation (Fig. 6c). Representative immunocytochemistry photomicrographs of all exposure experiments described above are shown in Fig. 6d.

Lastly, astrocytes expressing human α-synuclein with the A30P mutation, Tg(SNCA*A30P)Nbm, exposed to 0.5% TgM83-passaged MSA brain homogenate accumulated few α-synuclein inclusions (Additional file 1: Figure S8A,B). Astrocytes from this line exposed to brain homogenate from a TgM83^+/+^ control littermate (blue circles) or unexposed control cells (grey circles) did not exhibit phosphorylated α-synuclein accumulation (Additional file 1: Figure S8B). Absence of inocula carryover in our cell culture model system was confirmed by the lack of α-synuclein immunostaining in astrocytes isolated from the *Snca*^0/0^ line exposed to 0.5% TgM83-passaged MSA or TgM83^+/+^ control littermate brain homogenates, or exposed to 10 μg/mL recombinant α-synuclein fibrils (Additional file 1: Figure S8C). The kinetics of α-synuclein inclusion formation in all of the Tg astrocyte cultures are summarized in Table 1.

**Table 1:** MSA prion infection of four different Tg mouse astrocyte cultures expressing human α-synuclein

Astrocyte donor mice	Human α-synuclein levels relative to mouse^a^	Mouse α-synuclein	Relative rank of human α-synuclein levels^b^	pSyn (S129) intensity after exposure to MSA patient–derived or [TgM83-passaged] brain homogenate^c^			
				0 dpe	7 dpe	14 dpe	21 dpe
Tg(SNCA*A53T)M83^+/+^	4.6x +/- 0.8*	Yes	++++	1[0.5]	5.7[2.9]	14.2 [8.9]	23[13.8]
Tg(SNCA*A53T)M83^+/–^	3.3x +/- 0.5*	Yes	+++	1[0.5]	2[0.6]	6.7[3.3]	10.7[6.3]
Tg(*SNCA*^+/+^)Nbm	1.3 – 2x^§^	No	+++	1.1[0.7]	3.4[1.5]	11[10.5]	18.7[22.0]
Tg(SNCA*A53T)Nbm	1.3 – 2x^§^	No	++	1[0.6]	2.2[0.6]	9.6[2.3]	12.9[3.5]
Tg(SNCA*A30P)Nbm	1.3 – 2x^§^	No	+++	0.9[0.5]	1.4[0.6]	3.7[2.9]	6.7[3.5]
*Snca* ^0/0^	None ^¥^	No	-	0[0]	0[0]	0[0]	0[0]

^a^Levels of human α-synuclein in brain cortex relative to levels of endogenous mouse α-synuclein—* [28]; ^§^ [41]; ^¥^ [10].

^b^mAb Syn211 was used to estimate human α-synuclein levels in cultured Tg astrocytes (20 μg of total protein analyzed by Western blot).

^**c**^mAb pSyn (S129) staining intensity in MSA-infected Tg astrocytes. Cultures were exposed to either 0.5% MSA_35_ patient–derived or 0.5% mouse-passaged MSA [(MSA_2_→TgM83) →TgM83] brain homogenate (values in brackets). Data represent the fluorescent signal intensity of phosphorylated α-synuclein (S129) immunostaining per cell (x10^4^) summarized from experiments described in Figs. 1, 2, 6, and S8. Details of data acquisition and analysis are described in the Material and Methods. Note: The initial amounts of MSA prions in the two different inocula were not normalized

### Astrocytes accumulating MSA prions do not induce obvious retraction of neuronal dendritic spines

**Fig. 7:**
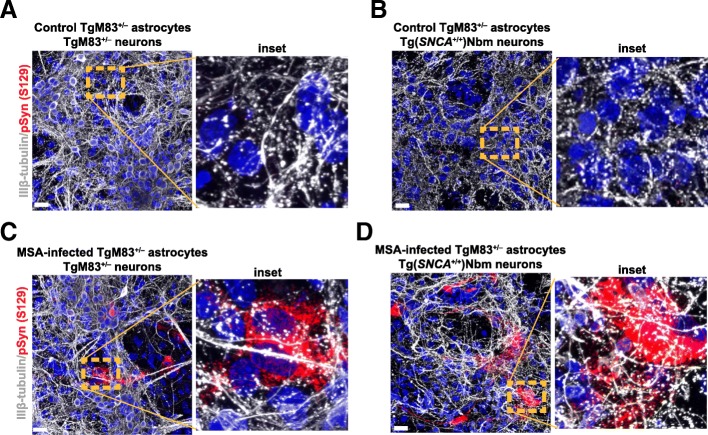
Astrocyte cultures infected with MSA prions do not show retraction of neuronal dendritic spines. TgM83^+/−^ astrocytes were infected with MSA prions and maintained in an FBS-free medium for 21 days. Freshly isolated neuronal cultures from either (**a**, **c**) TgM83^+/−^ or (**b**, **d**) Tg(*SNCA*^+/+^)Nbm P0 mice were plated on a layer of (**a**, **b**) control or (**c**, **d**) MSA-infected astrocytes. Cells were then cultured in FBS-free medium for 14 days. Immunostaining was carried out with pSyn (S129) (red) to reveal MSA prion infection and IIIβ-tubulin (white) to assess neuronal integrity. Insets of dashed areas are shown on right. Nuclei were stained with DAPI (blue). Scale bars, 50 μm

A report by Harris and colleagues suggested that PrP^Sc^ prions cause synaptotoxicity [24]. We, therefore, investigated the effect of MSA prions on the integrity of dendritic spines using differentiated cultures of neurons from mouse frontal cortex. Neurons from TgM83^+/−^ or Tg(*SNCA*^+/+^)Nbm mice were plated on a layer of astrocytes, either control (Fig. 7a,b) or MSA-infected at 21 dpe (Fig. 7c,d), and maintained in vitro for 14 days. Cells were then analyzed by immunocytochemistry. We did not observe retraction of neuronal dendritic spines in the neurons cultured with MSA-infected astrocytes (insets, Fig. 7c,d). Additionally, we did not detect abnormal cell death in cultures with α-synuclein inclusions compared with those exposed to control brain homogenate or unexposed control cells (Additional file 1: Figure S9A–D).

## Discussion

Because astrocytes and oligodendrocytes express less α-synuclein than neurons [59, 60], the origin of GCIs in MSA remains ambiguous. It is unclear whether GCIs are a consequence of a primary glial α-synucleinopathy followed by neurodegeneration or an α-synucleinopathy leading to inclusions within glia [64, 101]. In the studies reported here, we show that primary astrocytic cultures isolated from Tg mice overexpressing wt or mutant human α-synuclein can rapidly accumulate α-synuclein inclusions after exposure to MSA patient brain homogenates. Our findings substantiate recent reports that α-synuclein inclusions accumulate in the astrocytes of α-synucleinopathy patients [8, 19, 30, 62, 95].

### Exogenously added recombinant α-synuclein fibrils induce aggregation and phosphorylation of endogenous human α-synuclein in Tg astrocytes

Astrocytes readily take up extracellular recombinant α-synuclein in vitro [12, 25, 44, 76], and Tg mice inoculated with α-synuclein fibrils have also shown formation of α-synuclein inclusions in astrocytes [29, 46, 52, 78, 79, 82]. However, it is likely that the promoter driving α-synuclein expression dictates cell tropism of inclusion formation in these models. TgM83 mice express human α-synuclein with the A53T mutation under control of the heterologous *Prnp* promoter. It has been previously established that PrP is expressed in astrocytes [34, 38, 39, 48, 61, 92], and both neurons and astrocytes are sites of PrP prion replication and accumulation [2, 38, 75]. Accordingly, we have shown that endogenous transgene-expressed α-synuclein in TgM83 astrocytes undergoes phosphorylation and forms inclusions when exposed to α-synuclein fibrils. We found that exogenously added artificial α-synuclein fibrils efficiently induced dose- and gene-dose-dependent aggregation and phosphorylation of endogenously expressed α-synuclein.

### Cultured astrocytes from Tg mice expressing human wt or mutant α-synuclein propagate MSA prions and form inclusions after exposure to MSA brain homogenate

We used patient-derived or mouse-passaged brain homogenate to propagate MSA prions. Here, aggregation and phosphorylation of endogenous α-synuclein is initiated rapidly and increases over time in GFAP-positive TgM83 astrocytes exposed to MSA brain samples. Similar rates of α-synuclein inclusion formation were observed after astrocytes were exposed to either human or mouse-passaged MSA brain tissue. Moreover, we have successfully passaged MSA prions into naïve cultures of TgM83 astrocytes. In all cases of MSA prion exposure, α-synuclein inclusion formation was dependent on prion dose and level of expressed α-synuclein.

This finding agrees with previous reports [28, 99]. TgM83^+/+^ mice develop spontaneous disease at 8 to 16 months, while TgM83^+/−^ mice develop spontaneous disease after 22 months of age [28]. MSA prion–inoculated hemi- or homozygous TgM83 mice harbor α-synuclein aggregates in hindbrain neurons and succumb to hindlimb paralysis with incubation times reflecting α-synuclein expression levels [80, 99, 102].

In the three additional Tg mouse lines we investigated, inclusion formation was most robust in the MSA-infected Tg(*SNCA*^+/+^)Nbm astrocytes exposed to either patient-derived or mouse-passaged MSA brain homogenate. MSA-exposed Tg(SNCA*A53T)Nbm and Tg(SNCA*A30P)Nbm astrocytes did not accumulate appreciable levels of inclusions. Notably, all three lines exhibited signs of enteric nervous system dysfunction and motor function abnormalities in vivo without neurological illness after inoculations with either α-synuclein fibrils or MSA brain homogenate [3, 9, 10, 41]. Analogously, inoculating TgM83 mice with MSA brain homogenate caused severe neurological disease. Of note, the in vitro expression of α-synuclein in neuronal cells isolated from Tg(*SNCA*^+/+^)Nbm mice was much lower than in neurons isolated from the TgM83^+/−^ line (data not shown). This finding points to our earlier observation that differences in the expression profile of α-synuclein or the promoter driving expression likely affect cell tropism and the accumulation pattern of α-synuclein pathology within the brain.

The number of inclusions formed in MSA-exposed TgM83^+/+^ and Tg(*SNCA*^+/+^)Nbm astrocytes in vitro was similar. This can be accounted for by the fact that despite the slightly lower α-synuclein expression in astrocytes from the latter line, the sequence of α-synuclein in MSA brain homogenate is wild-type, which is the same as the α-synuclein expressed in the Tg(*SNCA*^+/+^)Nbm astrocytes on the mouse *Snca* knockout background. In contrast, TgM83 mice express the PD-linked A53T mutation on a mouse *Snca* wt background, whose residue 53 also is threonine; therefore, in addition to human α-synuclein (A53T), endogenous murine α-synuclein might also be recruited into aggregates. Overall, we found that efficiently inducing α-synuclein aggregation and inclusion formation depends on (1) the promoter driving α-synuclein expression and (2) the amount of available endogenous α-synuclein available in the cell. This suggests that the cell types expressing α-synuclein and threshold of protein levels may be the determining factors for ensuring efficient MSA prion–induced α-synuclein pathology.

### MSA is a prion disease

It is widely accepted that prions are proteins that acquire alternative conformations that self-propagate [73]. Similar to PrP^Sc^ prions, α-synuclein was recently found to assemble into β-sheet-rich amyloid fibrils [31, 66, 67, 74, 99].

α-Synuclein is thought to occur in at least two structural isoforms: a natively unfolded monomer and a helix-rich membrane-bound form. These isoforms can undergo structural changes resulting in the formation of β-sheet-rich assemblies. Additionally, α-synuclein has been proposed to exist in a dynamic equilibrium. Its monomer can first aggregate into several types of small oligomeric species that can be stabilized by β-sheet interactions. These species can then form into higher-molecular-weight insoluble protofibrils that can polymerize into amyloidogenic fibrils resembling those found in Lewy bodies [5, 6, 20, 97]. We have shown that exposing cultured astrocytes to MSA patient brain homogenates initiates α-synuclein aggregation and phosphorylation, resulting over time in the formation and spread of α-synuclein inclusions. This further demonstrates that MSA is a prion disease.

In the early stages of inclusion formation, there appears to be a greater abundance of unphosphorylated aggregated total α-synuclein and a lower amount of phosphorylated α-synuclein. However, by the time aggregates reach > 50 μm in size, the majority of aggregated α-synuclein is phosphorylated. Our finding agrees with the observation that a majority (> 90%) of α-synuclein in the brains of α-synucleinopathy patients is phosphorylated at the S129 residue [1, 27].

The five α-synuclein phosphorylation sites identified experimentally are S87, Y125, S129, Y133, and Y136 [13, 14]. The physiological relevance of S129 phosphorylation is still unclear; however, it is thought to have profound effects on protein trafficking [55]. The C-terminus of α-synuclein can be phosphorylated at Y125 as well [21, 63, 65], as also observed in our MSA-infected astrocytes, and it has been proposed that phosphorylation of α-synuclein at Y125 and S129 have opposing effects on neurotoxicity and soluble oligomer formation [14].

### Filamentous and granular morphology of α-synuclein inclusions form in MSA-infected astrocytes

Different morphologies of α-synuclein aggregates are thought to represent different strains of α-synucleinopathies [56, 69, 70, 81]. Here, we report that the same MSA brain homogenate can induce two morphologically distinct inclusions in exposed astrocytes. In TgM83 astrocytes expressing human α-synuclein (A53T) on a murine *Snca* wt background, two-thirds of the inclusions were filamentous and one-third were granular. Interestingly, in Tg(*SNCA*^+/+^)Nbm astrocytes expressing human wt α-synuclein on a mouse *Snca* knockout background, α-synuclein inclusions were solely filamentous. The form of α-synuclein inclusion, therefore, may be dictated by intracellular milieu or α-synuclein posttranslational modifications rather than being reflective of prion strains.

### Astrocyte involvement in the pathology of α-synucleinopathies

The precise identity of the toxic species of α-synuclein remains controversial [14], and the cellular pathogenic mechanisms that underlie neurodegenerative processes in MSA are still poorly understood. In human PD brain tissue, the number of large intracellular aggregates generally correlates with neuronal dysfunction and disease severity [33]. In vivo and in vitro experimental studies have shown conflicting results with respect to the nature of the toxic species of α-synuclein [43, 47]. Intriguingly, exposure to LBD patient brain extracts has no effect on glutamatergic neurons but a detrimental effect on GABAergic neurons, which undergo dramatic inhibition of neurite outgrowth [12]. However, electron microscopic studies of PD patient brain have notably revealed well-preserved organelles in inclusion-bearing nigral neurons, suggesting that some neurons bearing inclusions remain relatively healthy [26].

Our study provides compelling evidence for the formation of phosphorylated α-synuclein inclusions in MSA-exposed astrocytes overexpressing human α-synuclein; however, toxicity and loss of astrocytes within the 21 dpe time period analyzed in these studies were insignificant. Interestingly, we did not observe detrimental effects on neuronal growth or dendritic spines when neuronal cells were plated on MSA-infected astrocytes and co-cultured for two weeks. Moreover, the media from MSA-infected astrocyte cultures did not induce α-synuclein inclusion formation in naïve astrocyte cultures. This finding was surprising as α-synuclein toxic species were previously proposed to propagate via exocytosis or an exosome-mediated mechanism [22, 45, 85]. Similarly, oriented transfer of α-synuclein from neurons to astrocytes, but not vice versa, has been previously proposed [50]. This indicates that glia might engulf and scavenge aberrant α-synuclein species to protect neighboring cells from potential toxic events. It is plausible, however, that increased α-synuclein burden might impair the degradation machinery and initiate production of proinflammatory factors of the glial cell, thus creating a bidirectional feedback loop leading to reactivity, cytotoxicity, and cell death. It is worth mentioning that from our immunostaining observations (data not shown), small granular α-synuclein assemblies were localized to lysosomes; however, the large α-synuclein inclusions were clearly lysosome (LAMP1) negative and were too large and seemed to fill the majority of cell cytoplasm. Similarly, a recent study proposed that large amounts of oligomeric α-synuclein engulfed by astrocytes can negatively affect their lysosomal machinery, induce mitochondrial damage, and were found to be stored in the trans-Golgi network region [77].

Astrocytes respond to pathological stimuli by reactive astrogliosis, and reactive astrocytes are closely associated with α-synuclein pathology in human MSA or PD/DLB brains [8, 35, 58, 90] and in mouse models of MSA [74, 99]. Unfortunately, the main limitation of in vitro studies is that cultured astrocytes exhibit signs of reactivity even in the absence of a pathological stimulus (most likely due to the cytokine stimuli in the cell culture media). This precludes our ability to identify the underlying mechanisms of astrocyte reactivity and to understand disease etiology in α-synucleinopathies (such as MSA) that involve reactive gliosis. Moreover, senescent cells containing α-synuclein aggregates are thought to damage nearby cells [15, 87].

### Biochemical hallmarks of synucleinopathies are recapitulated in cultured astrocytes propagating MSA prions

The key hallmark of MSA is glial cytoplasmic inclusions (GCIs) composed of aggregated and phosphorylated α-synuclein. Moreover, GCIs share additional biochemical hallmarks of synucleinopathies, including co-localization with ubiquitin and p62 and positive amyloid labeling with FSB dye. Ubiquitination and p62 labeling are critical modifiers that tag proteins for degradation but are often associated with pathological protein deposits that are resistant to degradation [17, 36, 42, 51, 54, 83]. It has been proposed that α-synuclein phosphorylation alters macroautophagy responsible for eliminating larger protein structures, such as oligomers and aggregates [23, 88, 103]. Inhibition of autophagy leads to an increase in p62 proteins [4], as is also seen in cells affected by MSA. It has been proposed that the polyubiquitin-binding and homopolymerizing p62 protein may be involved in linking polyubiquitinated protein aggregates to the autophagic machinery and, therefore, may help clear aggregated proteins and reduce their toxicity [4, 89].

**Fig. 8:**
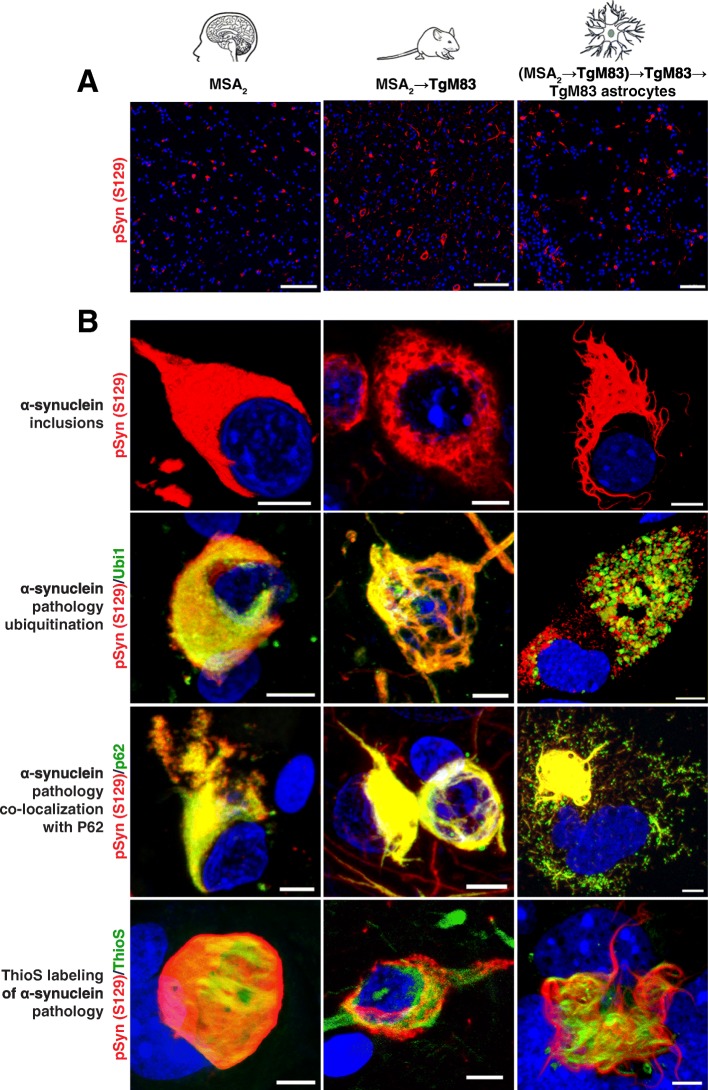
Hallmarks of synucleinopathies are recapitulated in cultured astrocytes infected with MSA. **a** Immunohistochemical detection of α-synuclein inclusions (red) in patient sample MSA_2_ (basal ganglia) (left), brain tissue from TgM83 mice inoculated with MSA_2_ (middle), and primary astrocyte culture infected with TgM83-passaged MSA_2_ prions. **b** Representative immunographs of high-power confocal microscopy of brain tissues and astrocyte culture from (**a**) immunostained for phosphorylated α-synuclein S129 (red) and ubiquitin (green; second row), p62 (green; third row), or amyloid detecting dye–FSB (green; fourth row). Nuclei were stained with DAPI (blue). Scale bars, (**a**) 200 μm, (**b**) 10 μm

We compared the pathological hallmarks of MSA in patient brain samples, brain tissue from TgM83 mice inoculated with MSA, and TgM83 primary astrocytes infected with MSA in vitro (Fig. 8). Low-magnification microscopy showed that phosphorylated α-synuclein inclusions (red) were similarly distributed in humans, mice, and astrocytes (Fig. 8a). Furthermore, high-power confocal microscopy confirmed that aggregated and phosphorylated α-synuclein inclusions have juxtanuclear localizations and are ubiquitinated and co-localized with p62. However, although α-synuclein inclusions associate with the autophagy machinery, they may dampen its function resulting in inefficient degradation. Moreover, the inclusions were labeled with amyloid-binding dye, indicating they are comprised of amyloid fibrils formed by α-synuclein (Fig. 8b). Together, our findings argue that aggregates of phosphorylated α-synuclein might both resist and disrupt autophagic degradation, as has been previously suggested [98].

The primary astrocytic culture model described here recapitulates some of the key features of α-synuclein inclusions found in the brains of patients with MSA. Overall, this model provides the scientific community with the opportunity to elucidate how α-synuclein inclusions form and contribute to the pathogenesis of MSA, thus establishing a readily scalable system for facilitating drug discovery.

## Material and methods

### Mice

The Institutional Animal Care and Use Committee of the University of California, San Francisco, approved all animal procedures related to this study. All procedures were in line with the recommendations of the Panel on Euthanasia of the American Veterinary Medical Association and the National Institutes of Health publication, *Guide for the Care and Use of Laboratory Animals* [18].

Primary astrocyte cultures were generated from the following mouse lines: homozygous B6;C3-Tg (Prnp-SNCA*A53T)83Vle/J (abbreviated here TgM83^+/+^) mice purchased from The Jackson Laboratory (stock #004479, USA) overexpressing human α-synuclein with the A53T mutation under control of the *Prnp* promoter on a mixed C57BL/6xC3H background [28]. This line was bred with the C57BL/6xC3H F1 mice to generate the hemizygous TgM83^+/−^ mice. Further, three lines of Tg mice on a mouse *Snca* knockout background (*Snca*^*0/0*^) overexpressing human α-synuclein under the control of the P1 artificial chromosome (PAC) were a gift from Robert L. Nussbaum, University of California, San Francisco. Namely, FVB;129S6-Snca^*tm1Nbm*^Tg(SNCA)1Nbm/J (The Jackson Laboratory, stock #010710) abbreviated here Tg(*SNCA*^+/+^)Nbm, or carrying either the A53T or A30P mutations designated FVB;129S6-Snca^tm1Nbm^ Tg(SNCA*A53T)1Nbm Tg(SNCA*A53T)2Nbm/J (The Jackson Laboratory, stock #010799) and FVB;129S6-Snca^tm1Nbm^ Tg(SNCA*A30P)1Nbm Tg(SNCA*A30P)2Nbm/J (The Jackson Laboratory, stock #010788), abbreviated here Tg(SNCA*A53T)Nbm and Tg(SNCA*A30P)Nbm [10, 41].

### Human MSA and TgM83 mouse passaged MSA brain homogenates

Human brain tissues selected for the study were autopsy-proven and well-characterized cases of definite MSA. MSA_2_ isolate from the patient’s basal ganglia was previously described [99] and was obtained from the Parkinson’s UK Brain Bank at Imperial College London. MSA_35_ tissue (brain stem and occipital cortex) was obtained from the Stanford Alzheimer’s Disease Research Center. All human tissues had consent for research use.

The primary passage of MSA_2_ originated from successful experimental animal transmission studies using TgM83^+/−^ mice [99]. The second passage of MSA_2_ was generated by inoculation of TgM83^+/−^ mice with brain tissue from MSA_2_ inoculated TgM83. Additional mouse brain samples used in this study were derived from primary passage of MSA_6_ (substantia nigra) and MSA_7_ (basal ganglia) and have been previously described [74]. MSA animal transmission studies were designed as follows: human brain or mouse brain tissues were homogenized to 10% weight to volume (*w*/*v*) in sterile calcium and magnesium free Dulbecco’s phosphate-buffered saline (DPBS) (Gibco) and then diluted to 1% for inoculation using 5% (w/v) bovine serum albumin (BSA). Brain extracts were not sonicated before inoculation. Young TgM83^+/−^ mice were anesthetized with isoflurane at 8 weeks of age and then inoculated in the right parietal lobe with 30 μL of the 1% brain homogenate (∼30 μg of total protein) using a 27-gauge needle. Mice were checked daily for routine health and assessed two times a week for signs of neurological illness, in accordance with the standard diagnostic criteria for prion disease in mice [11]. Clinical onset of primary MSA passage was ∼100 days, and secondary passage was ∼90 days. Mice exhibited ataxia, circling behavior, weight loss, proprioceptive deficits, dysmetria, and paralysis, and were euthanized within 2 days of showing progressive CNS dysfunction. Brains were then removed and snap-frozen on dry ice and stored at − 80 °C. Each brain sample was homogenized in sterile DPBS and subjected to immunoblot analysis or fixed and analyzed by immunohistochemistry for the presence of phosphorylated α-synuclein. Control brain homogenate was obtained from uninoculated TgM83^+/+^ littermates generated by intercrossing of TgM83^+/−^ hemizygote mice and sacrificed at ∼5.5 months of age.

### Brain homogenate preparation

Brain tissue was first homogenized at 10% (w/v) in sterile DPBS/5% sucrose at 4 °C and then ribolysed for 40 s (FastPrep-24, MP Biomedicals) using ceramic lysis beads and matrix D tubes (FastPrep, MP Biomedicals). The homogenate was then cleared of particulate matter by centrifugation at 420×*g* for 20 s at 4 °C and aliquots were stored at − 80 °C.

### MSA-infected primary astrocyte lysate

Cell homogenate inoculum from MSA-infected astrocytes was prepared as follows: Astrocyte cultures at 21 dpe to MSA_2_ twice passaged TgM83 brain homogenate were washed twice with ice-cold DPBS, scraped in DPBS using a silicone cell scraper (USA Scientific), and homogenized using a 1 mL syringe with a 27-gauge needle at 4 °C. The cell homogenate was then cleared of particulate matter by centrifugation at 420×*g* for 20 s at 4 °C, aliquoted, and stored until further use at − 80 °C.

### Recombinant α-synuclein fibrils

Lyophilized powder of recombinant human full-length wt α-synuclein was diluted in water to a concentration of 20 mg/mL. Then 50 mM NaPi, pH 7.2, and 5 mM of NaCl were added to yield a final concentration of 150 mM. The recombinant protein at a final concentration of 5 mg/mL was then polymerized into fibrils in sealed 1.5 mL microfuge tubes under constant agitation (1000 rpm, in an Eppendorf Thermomixer comfort, Eppendorf AG, Germany) at 37 °C for 5 days. Quality control of the pre-formed fibrils was assessed by transmission electron microscopy, which identified the predominant α-synuclein aggregates as fibrils bundled together in dense arrays (data not shown). The NHS-ester Alexa Fluor 488 (A20000, Invitrogen, Carlsbad, CA, USA) was used for amine labeling of the α-synuclein fibrils according to the manufacturer’s instructions. Briefly, a solution of α-synuclein fibrils was incubated for 1 h at room temperature with Alexa Fluor 488 dye in a 1:1 protein/fluorophore molar ratio. The fibrils were then pelleted using tabletop centrifuge (Eppendorf AG, Germany) to remove any unbound dye. The supernatant was discarded; the pellet containing labeled fibrils was resuspended in DPBS. The centrifugation/resuspension step was repeated two times. Inoculum containing α-synuclein fibrils was prepared as follows: fibrils or Alexa Fluor 488–labeled fibrils were diluted in DPBS and sonicated for 5 min using water bath sonicator (Branson). Then the fibrils were diluted in cell culture medium to a desired concentration (2.5, 10, or 40 μg/mL).

### Primary cell cultures

Primary cultures of astrocytes were prepared from P1–P4 mouse brains. Cerebral cortices were dissected and dissociated in 15 mL tubes at 2 mg/mL papain (Worthington) in sterile Hybernate-A (Gibco) under agitation at 37 °C for 15 min. The papain was then inactivated with a mixture of ovomucoid solution and DNAase I (both Worthington). The cell suspension was then washed twice using pre-warmed Neurobasal media and centrifugation at 17×*g* for 5 min. The cell suspension was then strained through a 100 μm cell strainer (CellTreat), resuspended in Neurobasal medium supplemented with 10% (*v*/v) FBS and 10 U/mL penicillin and streptomycin (Gibco). Cells were then plated onto T-75 flasks (Falcon) pre-coated with poly-D-lysine at 5 μg/mL (Sigma) at around 10,000 cells per cm^2^. Cells were maintained in T-75 flasks at 37 °C in a humidified 5% CO_2_ chamber for 2 weeks before frozen down as stock for future cell culture experiments.

### Cell exposure regimen

Astrocytes were plated 1-week before experimental use at a density of 10,000 cells/well in 96-well μ-plates (Ibidi) pre-coated with poly-D-lysine and maintained in 10% FBS and 10 U/mL penicillin and streptomycin containing Neurobasal medium (unless stated otherwise). Cells were exposed to the inoculum for 48 h. The medium was then discarded, and cells were washed twice with 150 μl of DPBS/well and either fixed immediately at 0 days post-exposure (0 dpe) or further cultured in fresh (inoculum-free) medium up to 21 dpe. In the case of time course studies, cells were fixed, stored at 4 °C until the last time point was collected, and analyzed concurrently.

### Neuronal platedown on MSA-infected astrocytes

TgM83^+/−^ astrocytes were grown in FBS free media for 21 dpe to the MSA inoculum. Then freshly isolated primary cells from either the TgM83^+/−^ or the Tg(*SNCA*^+/+^)Nbm P0 mouse were plated on top of MSA-infected TgM83^+/−^ astrocytes. The primary neuronal-glial cells were obtained by the same procedure as primary cultures of astrocytes except the freshly isolated cells were strained through a 70 μm cell strainer (CellTreat) and resuspended in Neurobasal media supplemented with 1% GlutaMax, 0.5% B27 (containing retinoic acid) and 10 U/mL penicillin and streptomycin (all Gibco). The cells were then plated onto MSA-infected TgM83^+/+^ astrocytes at around 10,000 cells per cm^2^ and cultured for 14 days at 37 °C in a humidified 6% O_2_ and 5% CO_2_ chamber, with half media changes every 2 days, and then fixed for analysis.

### Primary antibodies

Human specific α-synuclein, clone Syn 211 (αSyn) (1:500, Thermo Fisher #AHB0261); anti-phosphorylated α-synuclein at serine 129 [pSyn (S129)] (1:1000, Abcam #ab51253); phosphorylated α-synuclein at tyrosine 125 (pSyn Y125) (1:250, Abcam #ab10789); anti-glial fibrillary acidic protein (GFAP) (1:2000, Abcam #ab4674); anti-glutamate aspartate transporter (GLAST) (1:250, Miltenyi Biotec #130-095-822); anti-ubiquitin (1:500, Thermo Fisher #13-1600); anti-p62/sequestosome 1 (p62) (1:1000, Abcam #ab56416); anti-lysosomal-associated membrane protein 1 (LAMP1) (1:1000, Abcam #ab25245); anti-microtubule associated protein 2 (Map 2) (1:5000, Abcam #ab5392); III β-tubulin (1:1000, Neuromics #MO15013); anti-ionized calcium binding adaptor molecule 1 (Iba1) (1:500, Wako, #019-19,741); β-actin loading control (1:5000, Thermo Fisher #PA1-183); the (*E,E*)-1-Fluoro-2,5-bis(3-hydroxycarbonyl-4-hydroxy) styrylbenzene (FSB dye) (Congo red derivative) (at 2.5 μM, Santa Cruz #760988-03-2).

### Immunocytochemistry

Cell cultures were washed twice with DPBS, then fixed with 4% (*w*/*v*) paraformaldehyde (Polysciences) for 10 min and permeabilized for 10 min with 0.1% (*v*/*v*) Triton X-100 (Thermo Fisher). The cells were then blocked with 3% (*w/v*) bovine serum albumin (Sigma) for 30 min, followed by incubation with the primary antibodies for 1 h. After three DPBS washes, the cells were incubated with secondary Alexa Fluor–conjugated antibodies (Invitrogen) for 1 h, followed by four DPBS washes. The nuclei were counterstained with DAPI (Invitrogen). The cells were then examined and images captured by confocal microscopy using the TCS SP8 (Leica) microscope using the Las X (Leica) imaging software or analyzed using a high-throughput screening IN Cell 6000 analyzer (GE Healthcare). All images from independent, but identical, experiments were acquired under the same conditions, and laser intensity levels were maintained constant throughout all experiments to reduce technical variability.

### Immunohistochemistry

Briefly, mouse brains were fixed with 10% (*v/v*) formaldehyde (Fisher Scientific) and then embedded in paraffin (Cardinal Health). Sections of 8 μm were cut and mounted on a glass slide (Leica). The sections were then deparaffinized with xylene followed by 100% alcohol and 95% alcohol and treated with 3% (*v/v*) H_2_O_2_ (Fisher Scientific) in MeOH (Fisher Scientific) for 30 min. Epitope retrieval was performed with 0.01 M citrate buffer (VWR) for 10 min using steam. Slides were then blocked with 10% (*v/v*) normal goat serum (Abcam) for 1 h and incubated with primary antibody diluted in 10% (*v/v*) goat serum overnight.

For bright field analysis, the bound antibody was detected using a Vectastain ABC peroxidase kit (Vector Laboratories cat#PK-4000) and visualized using 3,3-diaminobenzidine (Vector Laboratories cat# SK-4100). Slides were counterstained with hematoxylin (Fisher Scientific cat# SH26-500D) then differentiated with two time immersions in acid alcohol differentiation solution (Sigma Life Science cat# A3429-4 L) and imaged using an AxioScan.Z1 microscope (Zeiss).

For fluorescent analysis, the primary antibodies were detected using Alexa Fluor–conjugated secondary antibodies (Life Technologies). Sections were mounted using ProLong Antifade hard set mounting media (Thermo Fisher) and visualized using a Leica SP8 confocal microscope (Leica) or AxioScan.Z1 microscope (Zeiss).

### Immunoblotting

Samples of equal protein concentrations (20 μg of protein per sample) were supplemented with 4x NuPAGE lithium dodecyl sulfate sample buffer (Novex) at a final concentration of 1x and boiled at 100 °C for 10 min before being loaded onto a NuPAGE 4–12% Bis-Tris gel (Invitrogen) and subjected to electrophoresis for 45 min at 200 V using pre-set gel cassettes (Invitrogen) and NuPAGE MES-SDS 1x running buffer (Invitrogen). To determine molecular weight, a MagicMark™ XP Western protein standard (Invitrogen) and BenchMark prestained protein ladder (Invitrogen) were run alongside the samples. The gel was then electroblotted onto polyvinylidene difluoride (PVDF) membrane (Hybond-P, GE Healthcare) for 1 h at 30 V using 1x transfer buffer consisting of 4% 20x NuPAGE transfer buffer (Invitrogen), 16% MeOH, 80% distilled H_2_O. The PVDF membrane was then blocked with a solution of 5% (*w*/*v*) non-fat milk powder (ChemCruz) dissolved in TBS-T (200 mM Tris HCl, 150 mM NaCl, pH 7.6) containing 0.1% Tween 20 (Pierce) for 1 h and subsequently incubated with primary antibody in TBS-T for 1 h. Then a horseradish peroxidase-conjugated secondary antibody (Pierce) was incubated for 1 h. The membrane was developed using ECL Plus (GE Healthcare) and imaged using the ChemiDoc™ XRS+ System (Bio-Rad) following the manufacturer’s instructions.

### Quantification and statistical analysis

Cell culture exposures were performed in triplicate (technical replicates) and repeated on independently isolated batches of cells (experimental *n*) for each cell line, allowing for quantification and statistical analysis. An IN Cell 6000 analyzer was used to collect immunofluorescence data from 16 fields per well in each technical replicate. Images of all immunostained channels—total α-synuclein, phosphorylated α-synuclein (pS129), GFAP and DAPI—were captured. The data were analyzed using the IN Cell developer software utilizing an algorithm developed to identify intracellular phosphorylated α-synuclein aggregates within cellular structures positive for total α-synuclein. DAPI counterstaining was used to assess total cell count in each image. The signal intensity of α-synuclein aggregates per cell was normalized by the total cell count in the field. Data were plotted as a mean ± SD and analyzed in Prism v7.0 (GraphPad). Alternatively, in some cases, the cell count of each group (number of cells positive for a specific marker) was plotted as percentage of total cells. Data were acquired from 16 randomized fields from several experimental replicates (*n*) carried out in technical triplicate.

Quantification of filamentous and granular α-synuclein inclusions was performed as follows: Three average sizes of the phosphorylated α-synuclein inclusions were set: size I (≤10 μm), size II (10–50 μm), and size III (≥50 μm). The size was then automatically determined using Fiji’s particle counting plugin (National Institutes of Health) where size of aggregates (stage) was translated into a range of pixel values normalized by the cell count of the analyzed image, thus representing the percentage of each stage of phosphorylated α-synuclein aggregate in the analyzed image. Quantification of GLAST-expressing cells was performed as follows: the data were acquired from six randomized fields from three independent cell isolation experiments. The number of GLAST positive cells was normalized by total cell count and plotted as percentage of total cells.

## Additional files

Additional file 1: **Figure S1.** Isolation and characterization of astrocyte cultures from transgenic (Tg) mice. **Figure S2.** Human α-synuclein expression levels in astrocyte cultures from transgenic (Tg) mice. **Figure S3.** Exposure of TgM83 astrocytes to wt α-synuclein fibrils induces aggregation and phosphorylation of α-synuclein at serine 129**. Figure S4.** Inclusions in MSA-exposed astrocytes consist of aggregated α-synuclein. **Figure S5.** α-Synuclein inclusions form in TgM83 astrocytes exposed to TgM83-passaged MSA brain homogenate. **Figure S6.** Both filamentous and granular α-synuclein inclusions form in astrocytes expressing glutamate/aspartate transporter. **Figure S7.** p62 expression correlates with phosphorylated α-synuclein (S129) in MSA-infected TgM83 astrocytes. **Figure S8.** α-Synuclein inclusions form in astrocytes expressing α-synuclein with the A30P mutation but not in astrocytes from α-synuclein knockout mice. **Figure S9.** Accumulation of α-synuclein inclusions in MSA-infected astrocytes is not cytotoxic. (DOCX 2030 kb)
