# Supplementary material for: Replication of multiple system atrophy prions in primary astrocyte cultures from transgenic mice expressing human α-synuclein
Source: Acta Neuropathol Commun. 2019 May 20;7:81. doi: 10.1186/s40478-019-0703-9 (PMC6526619; doi:10.1186/s40478-019-0703-9)
Supplement: Supplementary file 1 — Figure S1. Isolation and characterization of astrocyte cultures from transgenic (Tg) mice. Figure S2. Human α-synuclein expression levels in astrocyte cultures from transgenic (Tg) mice. Figure S3. Exposure of TgM83 astrocytes to wt α-synuclein fibrils induces aggregation and phosphorylation of α-synuclein at serine 129. Figure S4. Inclusions in MSA-exposed astrocytes consist of aggregated α-synuclein. Figure S5. α-Synuclein inclusions form in TgM83 astrocytes exposed to TgM83-passaged MSA brain homogenate. Figure S6. Both filamentous and granular α-synuclein inclusions form in astrocytes expressing glutamate/aspartate transporter. Figure S7. p62 expression correlates with phosphorylated α-synuclein (S129) in MSA-infected TgM83 astrocytes. Figure S8. α-Synuclein inclusions form in astrocytes expressing α-synuclein with the A30P mutation but not in astrocytes from α-synuclein knockout mice. Figure S9. Accumulation of α-synuclein inclusions in MSA-infected astrocytes is not cytotoxic. (DOCX 2030 kb) [file 40478_2019_703_MOESM1_ESM.docx]

ONLINE RESOURCE


Supplementary Material:
Replication of multiple system atrophy prions in primary astrocyte cultures from transgenic mice expressing human α-synuclein


Acta Neuropathologica

Zuzana Krejciova^1^, George A. Carlson^1,2^, Kurt Giles^1,2^, and Stanley B. Prusiner^1,2,3*^

^1^Institute for Neurodegenerative Diseases, UCSF Weill Institute for Neurosciences, University of California, San Francisco, San Francisco, CA 94158; ^2^Department of Neurology, UCSF Weill Institute for Neurosciences, University of California, San Francisco, San Francisco, CA 94158; ^3^Department of Biochemistry and Biophysics, University of California, San Francisco, San Francisco, CA 94158

*Correspondence to: Stanley B. Prusiner, M.D., Institute for Neurodegenerative Diseases, University of California, San Francisco, Sandler Neurosciences Center, 675 Nelson Rising Lane, San Francisco, CA 94158, stanley.prusiner@ucsf.edu, https://orcid.org/0000-0003-1955-5498


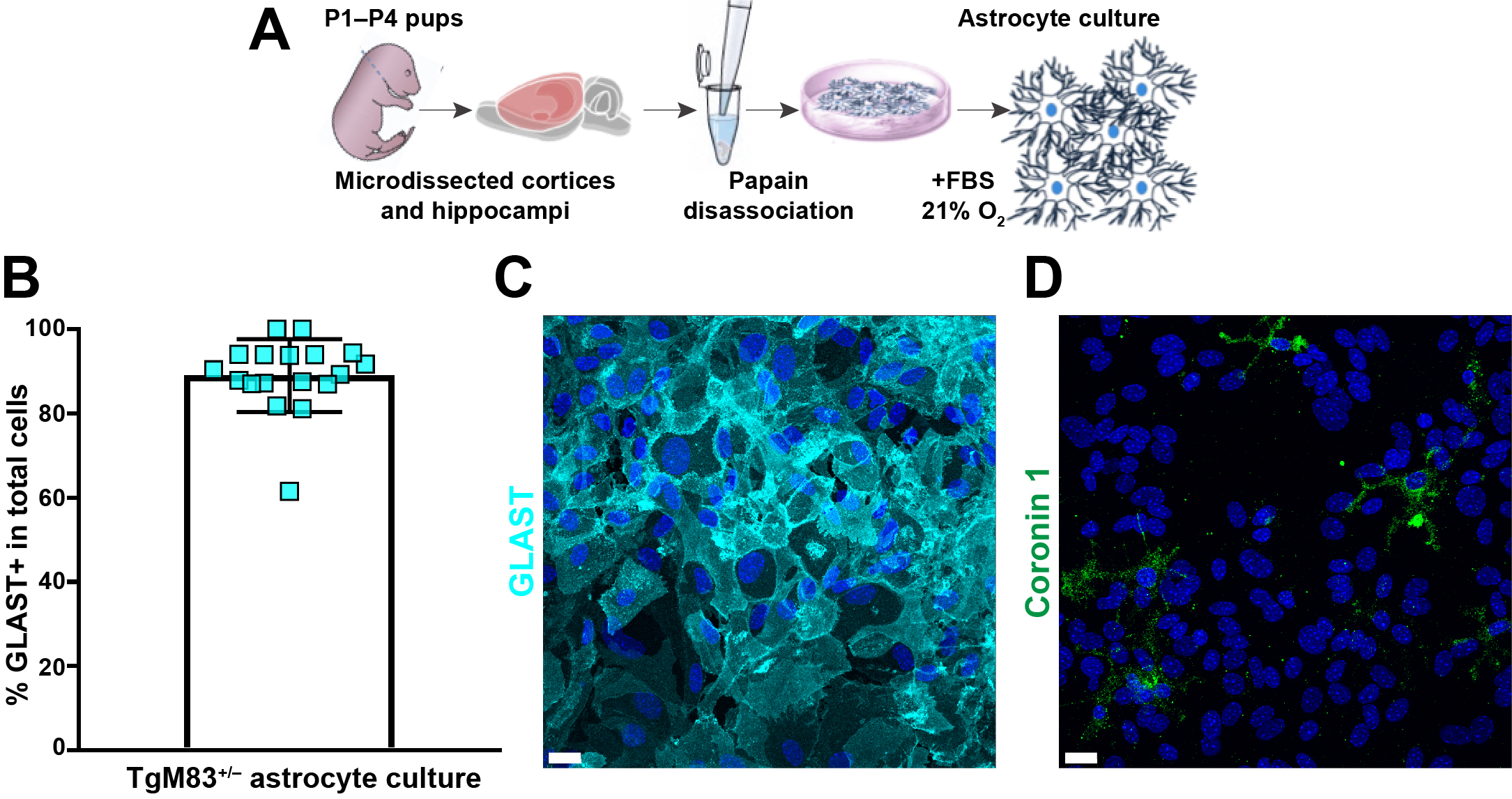


**Fig. S1: Isolation and characterization of astrocyte cultures from transgenic (Tg) mice.** (A) Schematic representation of primary astrocyte culture isolation from P1–P4 Tg mice. (B) Quantification demonstrating that ~ 90% of cells in our primary cultures were GLAST-expressing astrocytes. Representative immunograph of TgM83^+/–^ primary astrocyte cultures immunostained with (C) marker recognizing the extracellular epitope of the astrocyte-specific transmembrane glutamate-aspartate transporter (GLAST, cyan) and (D) anti-Coronin 1 (green) immunolabelling microglia. Nuclei were stained with DAPI (blue). Scale bars, 20 μm.

**
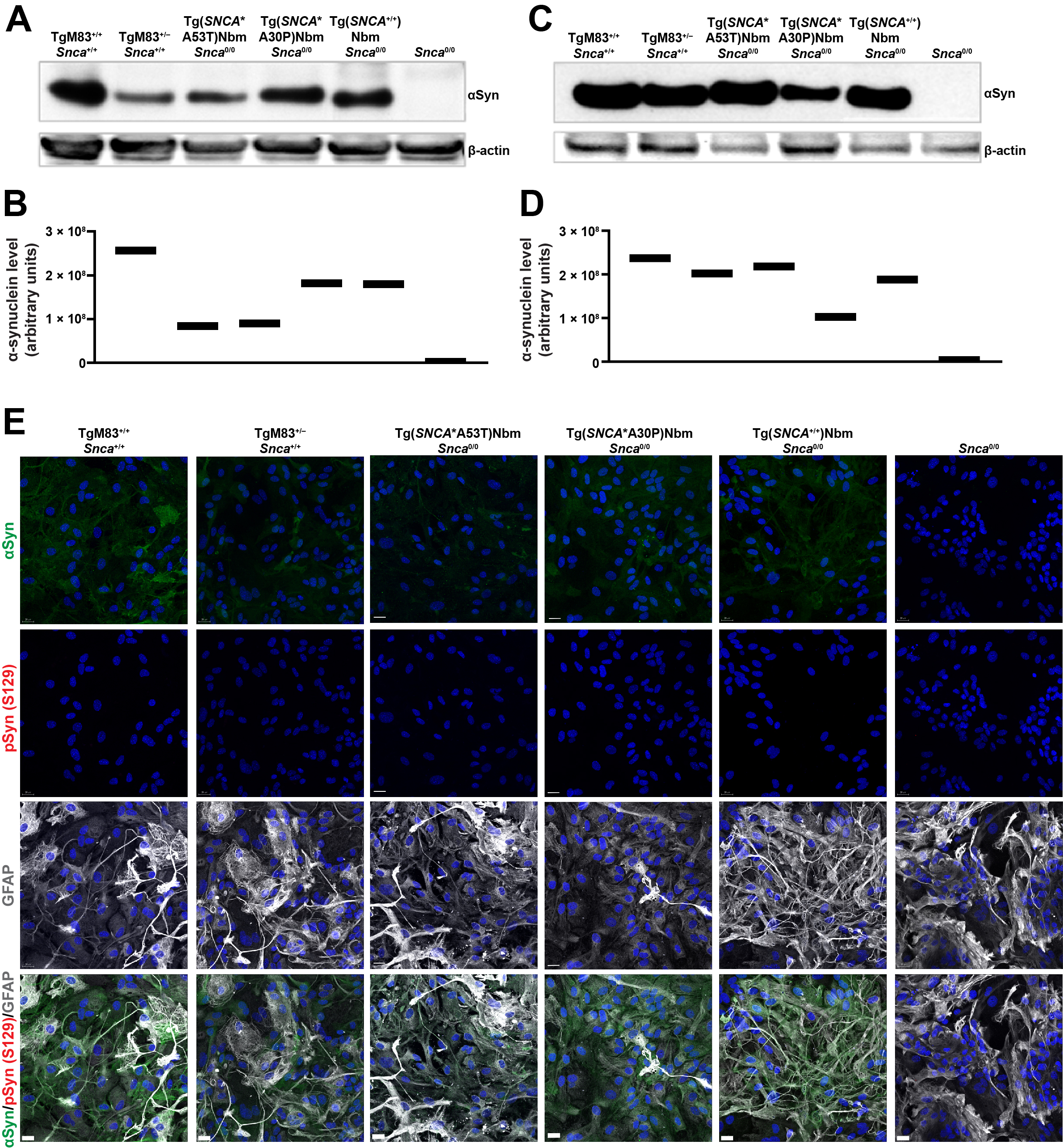
**

**Fig. S2: Human α-synuclein expression levels in astrocyte cultures from transgenic (Tg) mice.** (A) Representative immunoblots of human α-synuclein expression levels in primary cultures of astrocytes at 14 days *in vitro* and (B) graphic representation of (A). (C) Representative immunoblots of human α-synuclein expression levels in whole brain homogenates from P1–P4 Tg mice and (D) graphic representation of (C). (A,C) The amount of total protein loaded was 20 μg/lane. The blots were developed with anti-α-synuclein Syn211 antibody and then stripped and reprobed with anti–β actin antibody as a loading control. (E) Representative immunographs of primary astrocytes of all lines at 40–45 days in vitro immunostained for total human α-synuclein (αSyn, green), α-synuclein phosphorylated at serine 129 [pSyn (S129), red], and glial fibrillary acidic protein (GFAP, white). Merge of all channels is shown in the bottom row. Nuclei were stained with DAPI (blue). Scale bars, 20 μm.


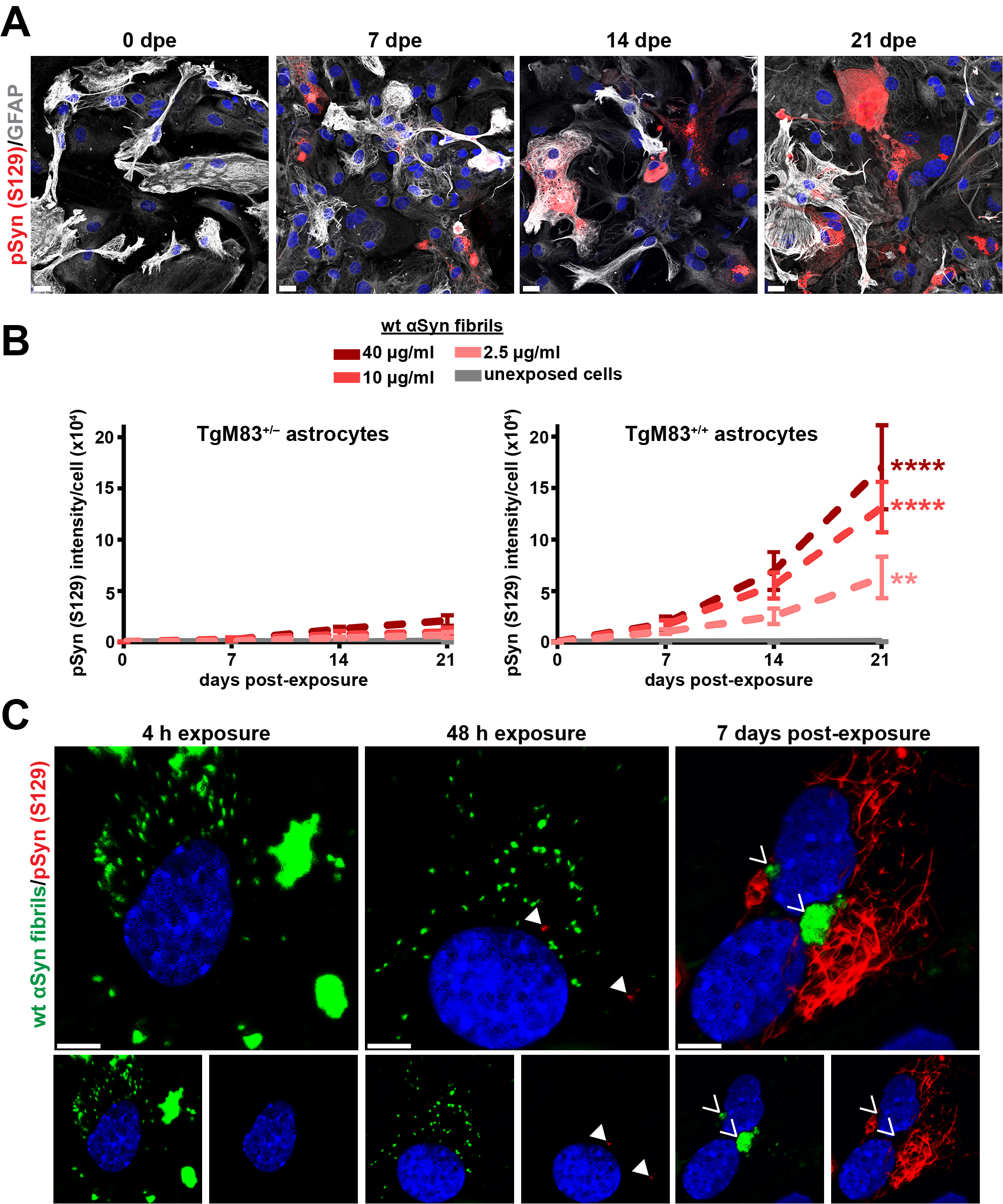


**Fig. S3: Exposure of TgM83 astrocytes to wild-type (wt) α-synuclein fibrils induces aggregation and phosphorylation of α-synuclein at serine 129.** (A) Representative immunographs of TgM83^+/+^ primary astrocytes exposed to recombinant wt α-synuclein fibrils at a final concentration of 10 μg/mL and immunostained for α-synuclein phosphorylated at serine 129 [pSyn (S129), red] and glial fibrillary acidic protein (GFAP, white) at 0, 7, 14, and 21 days post-exposure (dpe). Merge of the channels is shown. Scale bars, 20 μm. (B) Quantification of phosphorylated α-synuclein [pSyn (S129)] intensity in TgM83^+/–^ (left) and TgM83^+/+^ (right) astrocytes exposed to recombinant wt α-synuclein fibrils at a final concentration of 40 μg/mL (dark red), 10 μg/mL (light red), and 2.5 μg/mL (pink). The signal of phosphorylated α-synuclein was normalized by cell count. Data are plotted with mean ± SD (*n*=3 TgM83^+/–^, *n*=6 TgM83^+/+^, analyzed by one-way ANOVA, and followed by Tukey’s multicolumn comparison test: ****, *P* < 0.0001; **, *P* = 0.0024). (C) TgM83^+/+^ primary astrocytes were exposed to 10 μg/mL of Alexa Fluor 488–conjugated recombinant wt α-synuclein fibrils (green) for 4 h (left), 48 h (middle), or 48 h and recovered in fresh medium for 7 days (right). The cells were then immunolabeled for pSyn (S129) (red). Merge of channels (top) showing fibrils (green) and pSyn (S129) (red) and individual channels are shown (below). Small puncta of phosphorylated α-synuclein (red) are detected at the 48 h exposure time point (middle, full arrowheads). Fibrils (green) remain unphosphorylated (right, empty arrowheads). Nuclei were stained with DAPI (blue). Scale bars, 5 μm.

**
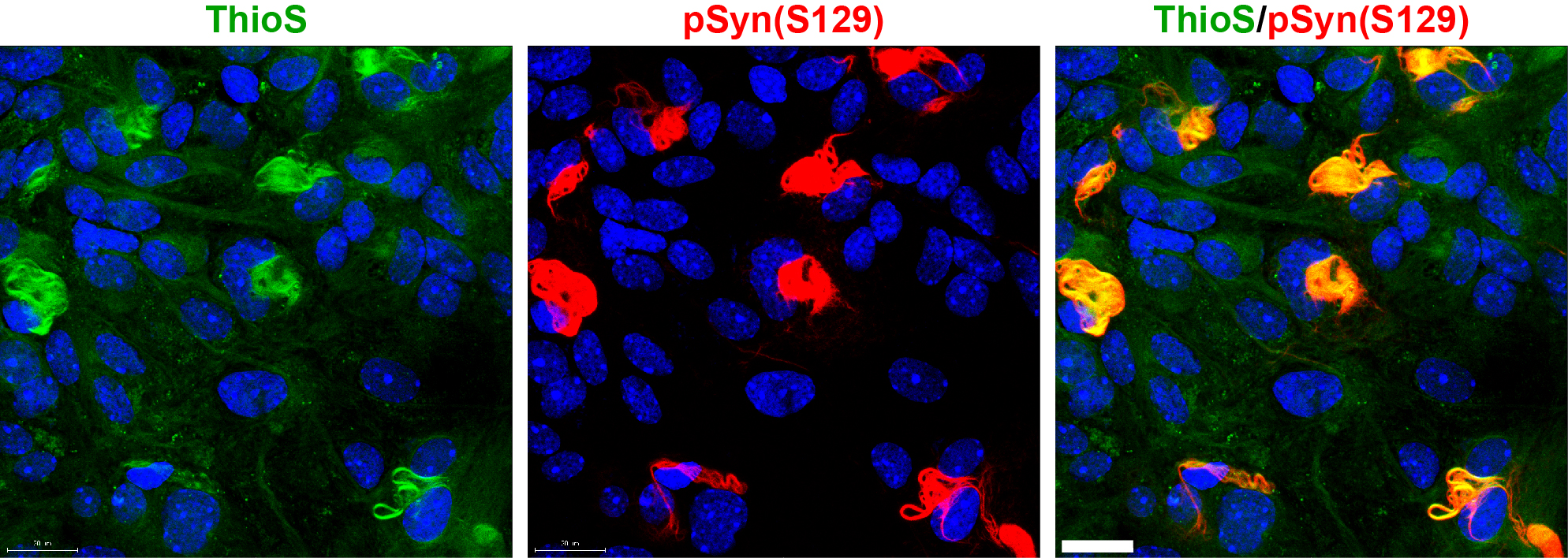
**

**Fig. S4: Inclusions in MSA-exposed astrocytes consist of aggregated α-synuclein.** Representative immunographs of TgM83^+/+^ primary astrocytes exposed to MSA brain homogenate and immunostained for amyloid detecting dye thioflavin S (ThioS, green) and phosphorylated α-synuclein [pSyn (S129), red] at 21 days post-exposure. Merge of channels is shown (right). Nuclei were stained with DAPI (blue). Scale bars, 20 μm.

**
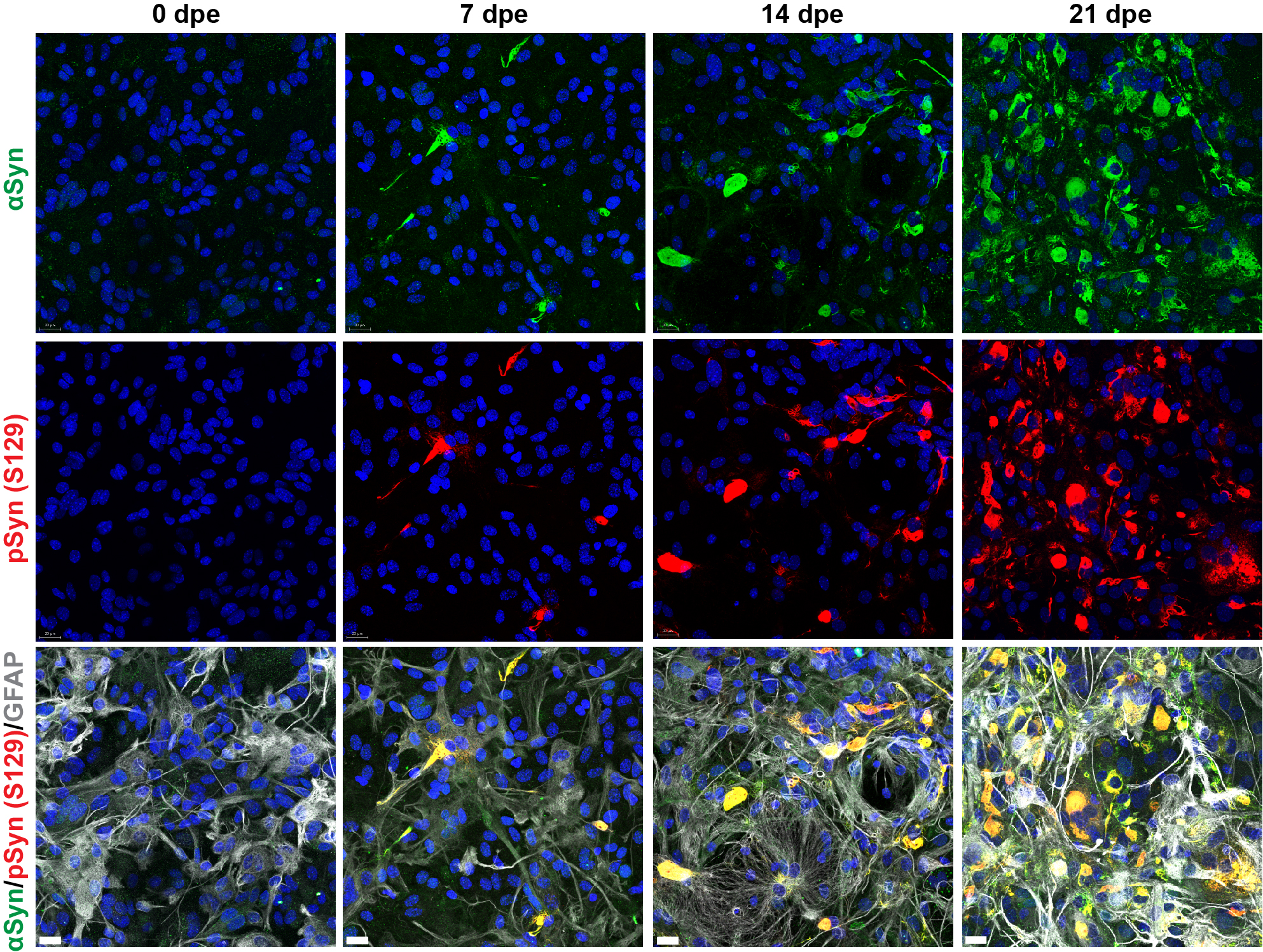
**

**Fig. S5: α-Synuclein inclusions form in TgM83 astrocytes exposed to TgM83-passaged MSA brain homogenate.** Representative immunographs of primary TgM83^+/+^ astrocytes exposed to 0.5% secondary passaged MSA_2_ brain homogenate for 48 h. Cultures were immunostained for total human α-synuclein (αSyn, green), pSyn (S129) (red), and glial fibrillary acidic protein (GFAP, white) at 0, 7, 14, and 21 days post-exposure (dpe). Merge of all channels is shown in the bottom row. Nuclei were stained with DAPI (blue). Scale bars, 20 μm.


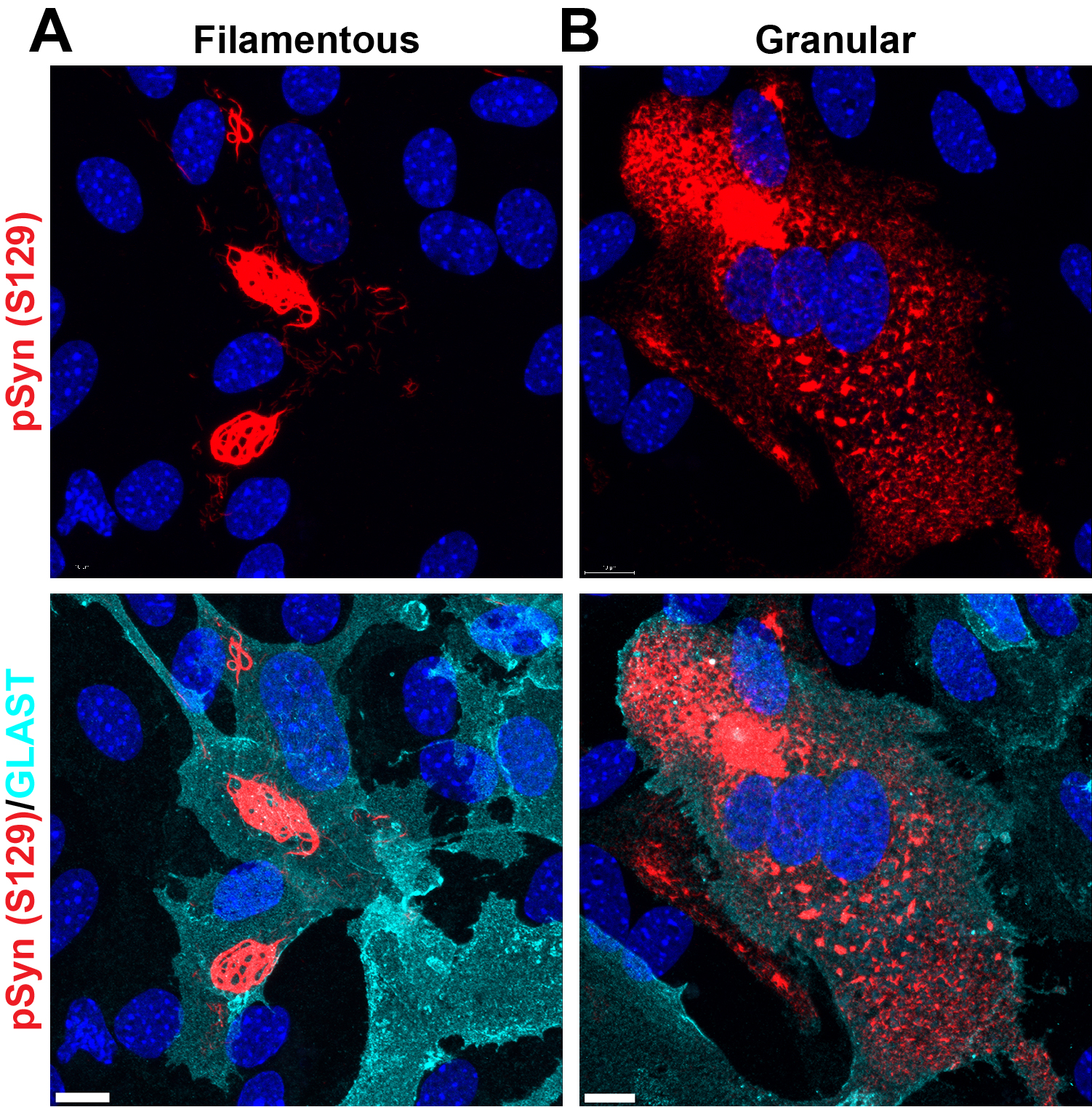


**Fig. S6: Both filamentous and granular α-synuclein inclusions form in astrocytes expressing glutamate-aspartate transporter.** TgM83^+/+^ astrocytes infected with MSA prions and immunostained for phosphorylated α-synuclein [pSyn (S129), red] and the extracellular epitope of the astrocyte-specific transmembrane glutamate-aspartate transporter (GLAST, cyan). Merge of channels is also shown in the bottom row. (A) Filamentous and (B) granular inclusions of aggregated α-synuclein are formed within astrocytic cytoplasm. Nuclei were stained with DAPI (blue). Scale bars, 10 μm.


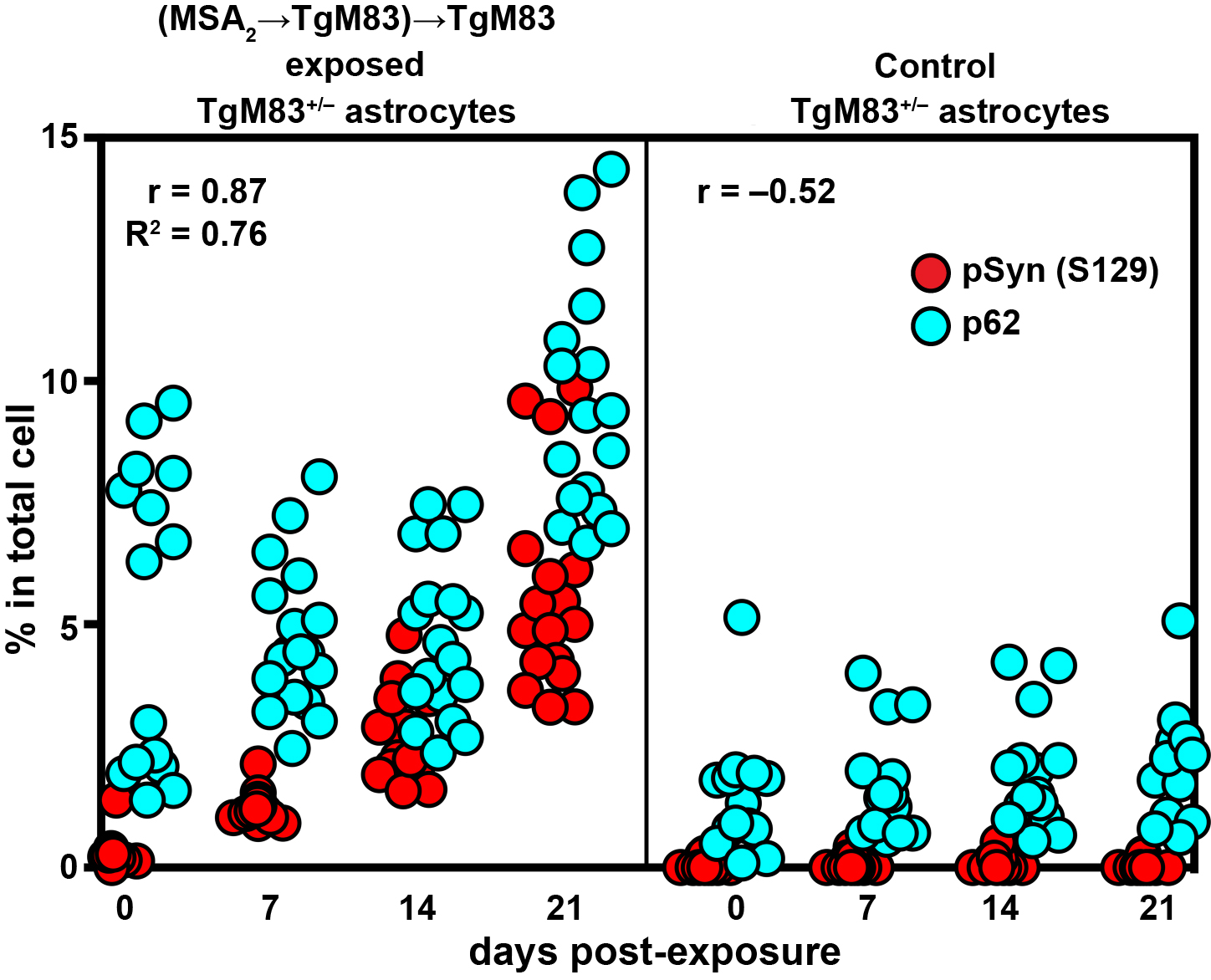


**Fig. S7: p62 expression correlates with phosphorylated α-synuclein (S129) in MSA-infected TgM83 astrocytes.** Quantification of TgM83^+/–^ astrocytes exposed to either 0.5% TgM83-passaged MSA_2_ brain homogenate (left) or unexposed as a control (right) and immunostained for pSyn (S129) (red) and p62 (turquoise) at 0, 7, 14, and 21 days post-exposure (dpe). Cell count of each group is represented as percentage of total cells where astrocytes containing signal of phosphorylated α-synuclein within p62 positive cells were counted. Data were acquired from 16 randomized fields from a technical replicate carried out in triplicate. Values of Pearson correlation coefficient of p62 and pSyn (S129) in astrocytes infected with MSA: r = 0.87 and R^2^ = 0.76, and in control unexposed cultures: r = –0.52.


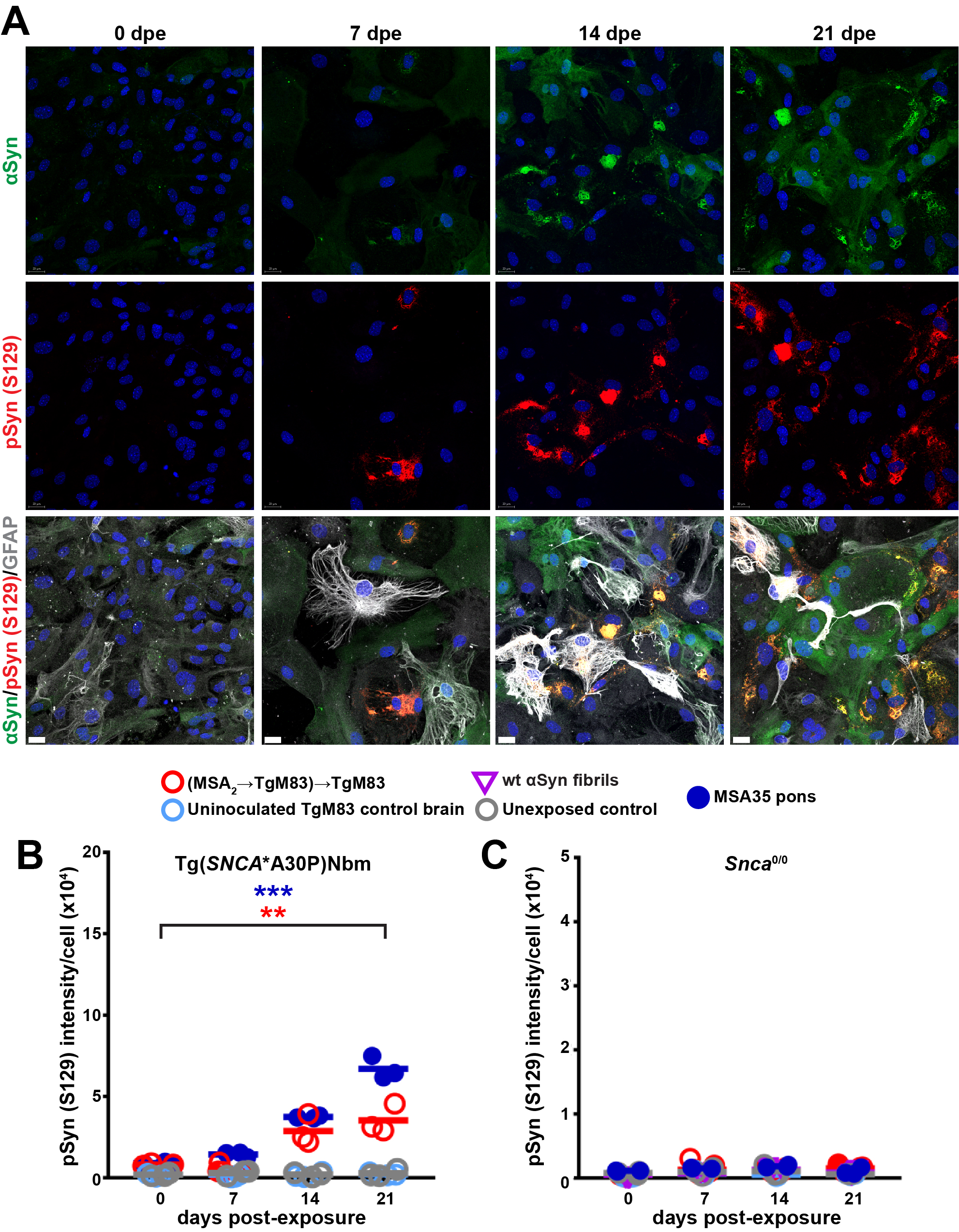


**Fig. S8: α-Synuclein inclusions form in astrocytes expressing α-synuclein with the A30P mutation but not in astrocytes from α-synuclein knockout mice.** (A) Tg(*SNCA**A30P)Nbm astrocyte cultures exposed to 0.5% TgM83-passaged MSA_2_ brain homogenate for 48 h and immunostained for αSyn (green), pSyn (S129) (red), and GFAP (white) at 0, 7, 14, and 21 days post-exposure (dpe). Merge of all channels is shown in the bottom row. (B) Quantification of pSyn intensity in Tg(*SNCA**A30P)Nbm astrocytes exposed to 0.5% brain homogenates from MSA_35_ patient (dark blue filled circles, *n*=3), TgM83-passaged MSA_2_ (red circles, *n*=3), age-matched TgM83^+/+^ littermate (light blue circles, *n*=3), and unexposed control cells (grey circles, *n*=3). (C) Quantification of *Snca*^0/0^ astrocytes exposed to brain homogenates as in (B) and recombinant wild-type α-synuclein fibrils at a final concentration of 10 μg/mL (purple triangles). (B,C) Data are plotted with mean (*n*=3) and analyzed by an unpaired *t*-test: ***, *P* = 0.001; **, *P* = 0.0063. Cultures were analyzed at 0, 7, 14, and 21 dpe. Astrocytes were immunostained for pSyn (S129), and the signal intensity was normalized by cell count. Data are plotted with mean. Nuclei were stained with DAPI (blue). Scale bars, 20 μm.


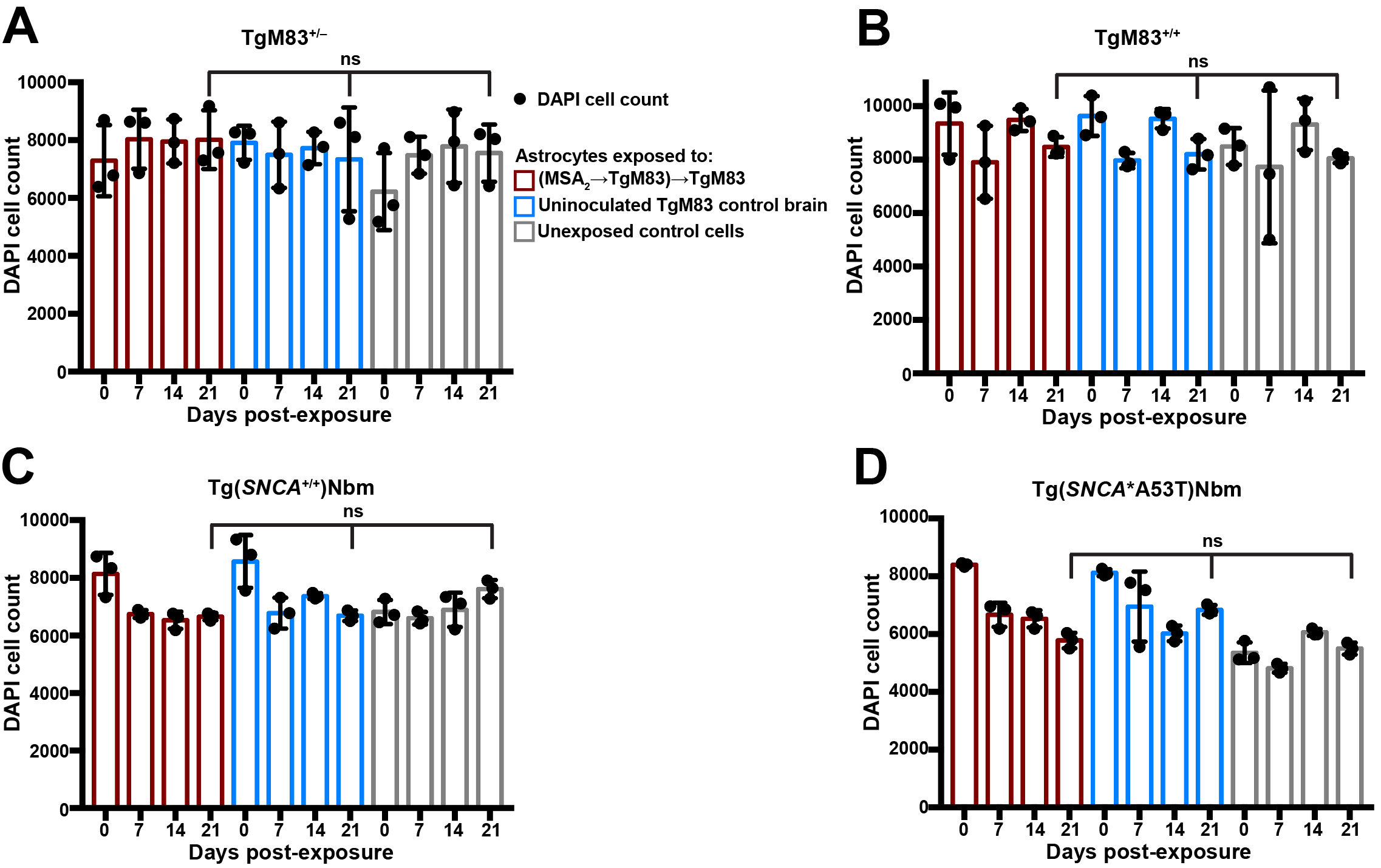


**Fig. S9: Accumulation of α-synuclein inclusions in MSA-infected astrocytes is not cytotoxic.** Quantification of cell count in cultures of astrocytes exposed to either second-passage MSA brain homogenate (red) or TgM83^+/+^ age-matched uninoculated control brain homogenate (blue), or unexposed cultures (grey) from (A) TgM83^+/–^, (B) TgM83^+/+^, (C) Tg(*SNCA*^+/+^)Nbm, and (D) Tg(*SNCA**A53T)Nbm mouse lines. Data are plotted with mean ± SD (*n*=3) and analyzed by one-way ANOVA followed by Tukey’s multicolumn comparison test: ns, not significant.
